# Epigenetic age acceleration mediates the association between pro-inflammatory and pro-oxidant diets and the progression and mortality of cardiovascular-kidney-metabolic syndrome

**DOI:** 10.1186/s12263-026-00804-0

**Published:** 2026-05-07

**Authors:** Shuang Wu, Siqi Lyu, Zhenkun Yang, Tianshu Gu, Yimeng Wang, Juan Wang, Jun Zhu, Yanmin Yang, Yang Chen, Lihui Zheng

**Affiliations:** 1 National Center for Cardiovascular Disease, Fuwai Hospital, Chinese Academy of Medical Sciences and Peking Union Medical College, Beijing, People’s Republic of China; 2 National Clinical Research Center of Cardiovascular Diseases, Fuwai Hospital, National Center for Cardiovascular Disease, Chinese Academy of Medical Sciences and Peking Union Medical College, Beijing, People’s Republic of China; 3 China-Japan Friendship Hospital (Institute of Clinical Medical Sciences), Chinese Academy of Medical Sciences & Peking union Medical College, Beijing, China; 4 Liverpool Centre for Cardiovascular Science at University of Liverpool, Liverpool John Moores University and Liverpool Heart and Chest Hospital, William Henry Duncan Building, 6 West Derby Street, Liverpool, L7 8TX UK; 5 Tianjin Key Laboratory of Ionic-Molecular Function of Cardiovascular Disease, Department of Cardiology, Tianjin Institute of Cardiology, The Second Hospital of Tianjin Medical University, Tianjin, People’s Republic of China; 6 Department of Cardiovascular and Metabolic Medicine, Institute of Life Course and Medical Sciences, University of Liverpool, Liverpool, UK; 7 Arrhythmia Center, Fuwai Hospital, National Center for Cardiovascular Diseases, Chinese Academy of Medical Sciences and Peking Union Medical College, No. 167, Beilishi Road, Xicheng District, Beijing, 100037 People’s Republic of China

**Keywords:** Cardiovascular-kidney-metabolic syndrome, Dietary inflammatory index, Dietary oxidative balance score, Epigenetic age acceleration, Oxidative balance, Mortality

## Abstract

**Background:**

The cardiovascular–kidney–metabolic (CKM) syndrome is a major public health challenge driven by intertwined cardiometabolic and renal dysfunction. Diet-related inflammation and oxidative stress may accelerate biological aging, as reflected by DNA methylation age acceleration, thereby contributing to CKM progression and mortality. However, these pathways have not been comprehensively examined.

**Methods:**

We analysed data from the National Health and Nutrition Examination Survey (NHANES) 1999–2002, including non-pregnant adults aged ≥ 20 years with complete dietary, epigenetic, and cardiometabolic data. Dietary inflammatory potential and antioxidant capacity were assessed using the Dietary Inflammatory Index (DII) and Dietary Oxidative Balance Score (DOBS), derived from 24-hour dietary recall data. DNA methylation age acceleration (DNAmAA) was quantified using multiple established epigenetic clocks. Cardiovascular–kidney–metabolic (CKM) syndrome was defined and staged according to contemporary criteria. Associations of dietary indices with DNAmAA, CKM stages, and all-cause and cause-specific mortality were examined using weighted regression and Cox proportional hazards models. Mediation analyses were performed to evaluate the role of DNAmAA in linking dietary patterns with CKM progression and mortality. All analyses accounted for the complex NHANES survey design and relevant confounders.

**Results:**

Participants with higher dietary inflammatory potential (higher DII) and lower antioxidant capacity (lower DOBS) exhibited less favourable sociodemographic and cardiometabolic profiles and more advanced CKM stages at baseline. Higher DII was consistently associated with accelerated epigenetic aging across multiple DNAmAA measures, whereas higher DOBS showed protective associations. Pro-inflammatory and pro-oxidative dietary patterns were associated with increased odds of advanced CKM stages and higher risks of all-cause and cardiovascular mortality, while anti-inflammatory and antioxidant dietary patterns were associated with lower risks. Mediation analyses demonstrated that GrimAge acceleration and DunedinPoAm partially mediated the associations of dietary indices with CKM progression and mortality, supporting a role for biological aging in linking diet-related inflammation and oxidative stress to adverse CKM outcomes.

**Conclusions:**

Dietary inflammatory and oxidative potential is associated with epigenetic aging, CKM progression, and mortality, partly mediated by GrimAge and DunedinPoAm. Improving dietary quality may represent a modifiable strategy to reduce CKM burden.

**Supplementary information:**

The online version contains supplementary material available at 10.1186/s12263-026-00804-0.

## Introduction

The Cardiovascular-Kidney-Metabolic (CKM) syndrome is a recently defined clinical construct that highlights the strong pathophysiological interplay and coexistence among cardiovascular disease (CVD), chronic kidney disease (CKD), and metabolic disorders such as type 2 diabetes and obesity [1, 2]. It refers to the concurrent presence of these conditions across a continuum ranging from early metabolic abnormalities to established cardiovascular disease, often categorized into progressive stages from risk factor accumulation (e.g., obesity and prediabetes) to subclinical and overt CVD. This syndrome represents a significant and growing public health burden worldwide, contributing to substantial morbidity and premature mortality [3, 4]. Individuals with CKM syndrome exhibit a heightened risk of progressive end-organ damage and fatal cardiovascular events, underscoring the urgent need to identify modifiable risk factors and underlying biological mechanisms that drive disease progression.

Diet is a key aspect of lifestyle that significantly influences the onset and progression of chronic diseases [5, 6]. Beyond its role in energy balance, diet can systemically impact health through its effects on chronic inflammation and oxidative stress [7]. The Dietary Inflammatory Index (DII) and the Dietary Oxidative Balance Score (DOBS) are two comprehensive measures designed to assess the overall inflammatory potential and antioxidant capacity of an individual’s diet, respectively [8, 9]. A higher DII score, indicative of a pro-inflammatory diet, and a lower DOBS, reflective of reduced antioxidant intake, have been independently associated with an increased incidence of CVD, CKD, and metabolic abnormalities [10–12]. However, their effects in the context of the multi-system CKM syndrome remain less explored.

Although aging is the primary risk factor for chronic diseases, chronological age is an imprecise proxy for biological health. Epigenetic clocks, particularly those based on DNA methylation (DNAmAge), provide a superior framework for quantifying the aging process. These biomarkers, derived from DNA methylation patterns, capture the cumulative impact of environmental exposures, lifestyle factors, and intrinsic physiological processes that influence an individual’s cellular condition, providing a more detailed assessment of biological aging [13–15]. Importantly, accumulating evidence suggests that epigenetic clocks are sensitive to lifestyle-related exposures, including dietary patterns, through mechanisms involving chronic inflammation, oxidative stress, and metabolic dysregulation [13, 16]. Diets with higher inflammatory and oxidative potential may influence DNA methylation profiles by altering immune signalling pathways, redox balance, and cellular senescence processes, thereby accelerating biological aging [17].

The evolution of DNAmAge estimators has progressed from first-generation clocks (e.g., HorvathAge, HannumAge) that predict chronological age, to subsequent models (e.g., PhenoAge, GrimAge, ZhangAge, LinAge) which are increasingly optimized to capture aging-related physiological decline and disease risk [18–20]. Notably, some of these second-generation clocks, such as GrimAge, incorporate components related to inflammation, metabolic function, and mortality risk, making them particularly relevant for studying the biological impact of diet and other lifestyle factors [21]. DNAmAge acceleration (DNAmAA), defined as the discrepancy between DNAmAge and chronological age, reflects an accelerated pace of biological aging and the cumulative burden of physiological stress and damage. DNAmAA has been established as a significant risk factor for multiple age-related diseases and mortality [22, 23]. In our previous study from the National Health and Nutrition Examination Survey (NHANES) data, DNAmAA was shown to be associated with CKM stages and mortality risk, highlighting its potential role as a biomarker of disease burden and prognosis [24]. However, the upstream lifestyle factors that may influence DNAmAA and contribute to CKM progression remain incompletely understood.

We therefore hypothesize that pro-inflammatory and pro-oxidative diets are associated with accelerated epigenetic aging, which in turn mediates the link between such dietary patterns and an elevated risk of CKM progression and mortality. To test this hypothesis, we analyzed data from NHANES to: (1) examine the associations of the DII and the DOBS, both individually and combined, with various DNAmAA measures; (2) assess the relationships between these dietary indices and CKM progression and mortality risk; and (3) assess the potential mediating role of DNAmAA in the relationship between diet and the progression and mortality of CKM.

## Methods

### Study population

The NHANES is a biennial survey conducted by the National Center for Health Statistics, aimed at assessing the health and nutritional status of the civilian population in the United States. This survey is conducted using a stratified multi-stage sampling method, with approximately 5,000 participants recruited annually from 15 counties. Comprehensive data are collected through face-to-face interviews, physical examinations, and laboratory assessments. The study received ethical approval from the Ethics Review Board of the National Center for Health Statistics, with all participants providing written informed consent.

Our study utilized data from NHANES conducted between 1999 and 2002 and involved non-pregnant adults aged 20 years and older who participated in the survey to ensure comparability and reduce potential confounding related to age-specific physiological differences. Individuals were excluded based on the following criteria: pregnancy or age < 20 years; missing information required for CKM staging; implausible total energy intake (< 500 kcal/day or > 8,000 kcal/day for men, and < 500 kcal/day or > 5,000 kcal/day for women), based on commonly used thresholds in nutritional epidemiology to reduce measurement error [25]; missing data for DII or DOBS components and covariates; or missing data for DNAmAAs. After applying these exclusion criteria, a total of 2,109 individuals were included in our study (eFigure 1).

All exposure variables (dietary indices), DNAm-based biomarkers, CKM components, and covariates were assessed at baseline during the same NHANES examination cycle (1999–2002), primarily at the Mobile Examination Center visit, ensuring temporal alignment across variables.

### Definition of CKM syndrome

CKM syndrome refers to the simultaneous presence of clinical or subclinical CVD, CKD, and metabolic disorders, with definitions based on previous research (eTable 1) [26–28]. Clinical CVD includes congestive heart failure, coronary heart disease, myocardial infarction, or stroke; subclinical CVD includes a predicted 10-year CVD risk of ≥ 20%. The 10-year CVD risk is calculated using a simplified American Heart Association algorithm that considers age, gender, smoking, blood pressure, cholesterol, diabetes, kidney function, and the use of anti-hypertensive drugs and statins (eTable 2) [29]. CKD risk assessment is based on the definition provided by Kidney Disease: Improving Global Outcomes, using estimated glomerular filtration rate (eGFR) thresholds (< 30, 30–44, 45–59, and ≥ 60 mL/min/1.73 m²) and urine albumin-to-creatinine ratios (< 30, 30–299, and ≥ 300 mg/g) for classification [30]. Metabolic disorders include conditions such as overweight/obesity, abdominal obesity, prediabetes, diabetes, hypertension, dyslipidemia, and metabolic syndrome. Participants were categorized into CKM stages based on the presence and combination of metabolic abnormalities, CKD, and cardiovascular disease, following a previously established framework. Briefly, stage 0 included individuals with normal metabolic profiles and no CKD or CVD; stage 1 included those with excess or dysfunctional adiposity without additional metabolic disorders; stage 2 included individuals with established metabolic risk factors or CKD; stage 3 represented subclinical CVD defined by a predicted 10-year CVD risk ≥ 20% or very high-risk CKD; and stage 4 included individuals with clinical CVD. Detailed criteria for each stage are provided in eTable 3.

### Assessment of DII, DOBS, and different combinations of DII and DOBS

All participants completed nutritional assessments via a 24-hour dietary recall, conducted by trained interviewers in a private room at the Mobile Examination Center, equipped with standardized measuring tools for accurate food intake reporting. The first 24-hour dietary recall was conducted during the Mobile Examination Center visit, which was the same visit during which blood samples and other clinical measurements were obtained in NHANES. To gain further insights into dietary habits, a follow-up phone interview was held 3 to 10 days later. Only data from the initial 24-hour dietary recall were used to calculate the DII and the DOBS, maximizing sample size and minimizing missing values. Detailed information on NHANES dietary interview procedures, U.S. Department of Agriculture coding, and Food and Nutrient Database for Dietary Studies-based nutrient derivation is available in the official NHANES dietary documentation.

The DII was derived from a scoring system developed by Shivappa et al., based on evidence from 1,943 studies examining 45 food parameters’ effects on six inflammatory biomarkers: interleukins (IL-1β, IL-4, IL-6, IL-10), tumor necrosis factor-α, and high-sensitivity C-reactive protein (CRP). Nutrients were assigned scores reflecting their inflammatory effects: +1 for pro-inflammatory, 0 for neutral, and − 1 for anti-inflammatory (eTable 4) [31]. The DOBS was calculated by summing scores for 16 nutrients associated with oxidative stress, categorized into prooxidants (e.g., total fat, iron) and antioxidants (e.g., dietary fiber, β-carotene, vitamins B2, B6, C, E, total folate, B12, calcium, magnesium, zinc, copper, selenium) as shown in eTable 5 [32]. A higher DII score reflects a greater dietary inflammatory potential, whereas a higher DOBS signifies a stronger antioxidant capacity.

Additionally, we integrated the DII and the DOBS to evaluate the combined effects of pro-inflammatory and pro-oxidative diets compared to anti-inflammatory and antioxidant diets on epigenetic age. Participants were categorized into three groups: those with diets high in pro-inflammatory and pro-oxidative components (third DII tertile and first DOBS tertile), those rich in anti-inflammatory and antioxidant components (first DII tertile and third DOBS tertile), and an intermediate group comprising participants not meeting either extreme.

### Measurement of DNAmAge

DNA was extracted from whole blood and stored at -80 °C for subsequent DNAm analysis. DNA methylation was assessed using the Zymo EZ DNA Methylation kit, which facilitated bisulfite conversion of 500 ng of DNA. The converted DNA was then hybridized to the Illumina Infinium Methylation EPIC BeadChip. After denaturation and overnight amplification, the BeadChip was washed and scanned with the Illumina iScan system. The resulting IDAT files were processed and normalized in RStudio, involving background subtraction, outlier detection, and imputation of missing values. The beta matrix was standardized, with outlier samples and probes removed to maintain data quality, and technical variation was adjusted using the ComBat method. This process produced several epigenetic biomarkers, including HorvathAge, HannumAge, SkinBloodAge, PhenoAge, GrimAge2Mort, ZhangAge, LinAge, WeidnerAge, VidalBraloAge, and DunedinPoAm. The preprocessing pipeline and derivation of DNAm-based biomarkers have been described previously in studies using the NHANES DNA methylation dataset and related epigenetic clock frameworks [33, 34]. Detailed descriptions of the DNA methylation preprocessing pipeline, normalization procedures, and derivation of epigenetic biomarkers are publicly available in the NHANES DNA methylation documentation (https://wwwn.cdc.gov/Nchs/Nhanes/DNAm/Default.aspx).

### Assessment of covariates

The study collected the following covariates: chronological age, sex, race and ethnicity, education level, poverty-to-income ratio (PIR), body mass index (BMI), systolic blood pressure (SBP), Healthy Eating Index (HEI)-2015 score, physical activity, smoking habits, alcohol consumption, cancer history, haemoglobin A1c (HbA1c), total cholesterol, high-density lipoprotein cholesterol, estimated glomerular filtration rate (eGFR), and C-reactive protein (CRP). The assignment scheme for the HEI-2015 score is provided in eTable 6.

### Definition of mortality outcomes

The primary outcome was all-cause mortality, and secondary outcomes included cardiovascular and non-cardiovascular mortality. Mortality status was ascertained by linking the NHANES database with the National Death Index via a rigorous probability matching process supplemented by death certificate review. The Linked Mortality Files incorporated follow-up data through December 31, 2019. Cause-specific mortality was classified according to the International Classification of Diseases, Tenth Revision (ICD-10).

### Statistical analysis

All statistical analyses were performed using R software (version 3.5.2; R Foundation for Statistical Computing). A two-tailed P-value < 0.05 was considered statistically significant.

To account for the complex, multistage sampling design of NHANES, sample weights, clustering, and stratification were incorporated in all analyses. Missing data (ranging from 0.05% to 11.14% across variables) were handled using multiple imputation with the ‘mice’ package in R (eTable 7). For descriptive statistics, continuous variables are summarized as mean ± standard deviation and compared across groups (DII tertiles, DOBS tertiles, and DII-DOBS combinations) using weighted analysis of variance, while categorical variables are presented as frequency (weighted percentage) and compared using weighted chi-square tests.

We used generalized linear regression models to evaluate the associations of the DII, DOBS, and their composite index with 10 DNAmAA measures (HorvathAA, HannumAA, SkinBloodAA, PhenoAA, GrimAA, ZhangAA, LinAA, WeidnerAA, VidalBraloAA, DunedinPoAm). All models were adjusted for chronological age, sex, race/ethnicity, PIR, smoking status, alcohol consumption, physical activity, HEI-2015 score, and CRP. To assess the associations between DII, DOBS, and the composite index with CKM stages, we used ordered logit regression models. The models were adjusted for confounders selected in advance, with three levels of adjustment: Model 1 accounted for chronological age, sex, and race/ethnicity; Model 2 added adjustments for PIR, smoking status, and alcohol consumption; and Model 3 included all covariates from Model 2, along with physical activity, HEI-2015 score, and CRP. Results are expressed as odds ratios (OR) with 95% confidence intervals (CI).

Cox proportional hazards regression models were used to examine the associations of dietary indices with all-cause, cardiovascular, and non-cardiovascular mortality, with adjustments for chronological age, sex, race and ethnicity, PIR, smoking, alcohol consumption, physical activity, HEI-2015 score, CRP, and history of cancer. Hazard ratios (HR) and 95% CIs are reported.

Two sensitivity analyses were performed to validate robustness: one excluding participants who died within the first 2 years of follow-up to minimize reverse causality, and another excluding participants with cancer, given that malignancy and its treatments may alter dietary patterns and are strongly associated with inflammation, oxidative stress, and mortality, thereby introducing potential residual confounding.

Mediation analysis was conducted to evaluate whether GrimAA and DunedinPoAm mediate the associations between dietary indices (DII, DOBS, DII-DOBS composite) and two outcomes: CKM stage progression, and all-cause/cardiovascular mortality in participants with CKM syndrome. A three-step adjusted regression framework was used, combined with a bootstrap method (500 resamples) to estimate 95% CIs for indirect effects. The confounders included in each step of the mediation analysis were consistent with those in the corresponding models for the respective outcomes: for the outcome of CKM stage progression, confounders were aligned with Model 3 of the ordered logit regression models; for the outcome of all-cause/cardiovascular mortality, confounders matched those in the fully adjusted Cox proportional hazards models. Step 1 regressed the mediator (GrimAA/DunedinPoAm) on the exposure (DII/DOBS) and the above confounders; Step 2 regressed the outcome (CKM stage/mortality outcomes) on the exposure and the same set of confounders; Step 3 regressed the outcome on the exposure, mediator, and the confounders. The proportion mediated was calculated as (indirect effect / total effect) × 100%, and an indirect effect with a bootstrap 95% CI not crossing zero was considered statistically significant.

## Results

### Baseline characteristics

Higher DII scores indicate a more pro-inflammatory diet, whereas higher DOBS values reflect greater antioxidant capacity and a more favorable oxidative balance. The baseline characteristics of participants, stratified by DII tertiles, DOBS tertiles, and their composite index, revealed significant differences in demographic and health-related variables (Table 1). Compared to those in lower DII tertiles, participants with higher DII scores showed a higher proportion of females and Non-Hispanic Black individuals, along with lower education and PIR levels and lower physical activity. They also exhibited higher smoking rates, more advanced CKM syndrome stages, and elevated HbA1c levels. Conversely, individuals in higher DOBS tertiles tended to be younger, Non-Hispanic White, and have higher education, PIR, HEI-2015 scores, and physical activity levels. Additionally, they had a lower proportion of current smokers, less advanced CKM syndrome stages, and better kidney function. When participants were grouped according to combinations of DII and DOBS, those in the anti-inflammatory and antioxidant diet group were younger, more male and non-Hispanic White, and had higher education levels compared with those in the pro-inflammatory and pro-oxidative diet group. Additionally, they demonstrated higher PIR, higher HEI-2015 scores, lower smoking rates, and less advanced CKM syndrome stages.

**Table 1:** Baseline characteristics of all participants categorized by DII tertiles, DOBS tertiles, and different combinations of DII and DOBS

Characteristics	DII				DOBS				Different combinations of DII and DOBS			
	Tertile 1 (< 0.87)	Tertile 2 (0.87-2.38)	Tertile 3 (≥ 2.38)	*P* value	Tertile 1 (< 10)	Tertile 2 (10-17)	Tertile 3 (≥ 17)	*P* value	Pro-inflammatory and pro-oxidative diet	Composite diet category	Anti-inflammatory and antioxidant diet	*P* value
Age, mean (SD), years	64.29 (0.45)	64.63 (0.56)	65.18 (0.64)	0.460	65.60 (0.49)	65.10 (0.65)	63.58 (0.41)	0.002	66.05 (0.63)	64.70 (0.46)	63.61 (0.50)	0.017
Male, n (%)	446 (57.97)	338 (44.45)	292 (33.21)	< 0.001	411 (46.78)	329 (44.17)	336 (46.56)	0.693	256 (39.12)	520 (44.85)	300 (52.51)	0.025
Ethnicity, n (%)				< 0.001				< 0.001				< 0.001
Non-Hispanic White	338 (85.15)	279 (76.73)	245 (74.90)		236 (71.82)	287 (79.20)	339 (85.03)		167 (71.18)	427 (78.64)	268 (86.13)	
Non-Hispanic Black	100 (4.78)	163 (10.04)	182 (11.70)		224 (14.46)	131 (8.06)	90 (4.61)		161 (14.31)	219 (8.94)	65 (3.97)	
Mexican American	204 (3.30)	190 (3.41)	214 (3.99)		241 (4.59)	186 (3.18)	181 (3.05)		176 (4.62)	284 (3.31)	148 (3.15)	
Hispanic and Other	61 (6.77)	71 (9.82)	62 (9.41)		63 (9.13)	71 (9.57)	60 (7.31)		49 (9.89)	99 (9.11)	46 (6.74)	
Education, n (%)				< 0.001				< 0.001				< 0.001
Less than high school	144 (6.00)	188 (10.93)	239 (15.57)		264 (15.89)	179 (10.17)	128 (6.77)		201 (17.68)	269 (10.24)	101 (5.88)	
High school or equivalent	237 (36.72)	280 (44.75)	317 (54.06)		328 (50.50)	262 (44.05)	244 (40.80)		242 (53.40)	409 (44.50)	183 (38.67)	
College or above	322 (57.29)	235 (44.32)	147 (30.37)		172 (33.62)	234 (45.78)	298 (52.43)		110 (28.92)	351 (45.26)	243 (55.44)	
Poverty income ratio, mean (SD)	3.48 (0.11)	2.94 (0.08)	2.62 (0.11)	< 0.001	2.57 (0.09)	3.14 (0.09)	3.30 (0.10)	< 0.001	2.40 (0.11)	3.07 (0.09)	3.44 (0.11)	< 0.001
BMI, mean (SD), kg/m^2^	28.39 (0.29)	29.08 (0.38)	29.07 (0.31)	0.103	28.75 (0.27)	29.15 (0.40)	28.61 (0.34)	0.470	28.96 (0.35)	29.14 (0.37)	28.21 (0.32)	0.133
SBP, mean (SD), mmHg	134.13 (1.05)	137.20 (1.37)	135.87 (1.19)	0.261	136.50 (1.18)	136.87 (1.11)	133.99 (1.27)	0.170	136.70 (1.42)	136.66 (1.02)	133.28 (1.22)	0.113
HEI-2015, mean (SD)	60.08 (0.68)	52.14 (0.73)	44.93 (0.70)	< 0.001	46.61 (0.78)	52.40 (0.83)	57.94 (0.80)	< 0.001	44.42 (0.88)	52.17 (0.64)	60.00 (0.82)	< 0.001
Physical activity, n (%)				< 0.001				< 0.001				< 0.001
Less than moderate	391 (46.22)	445 (52.09)	501 (67.18)		536 (64.91)	419 (53.78)	382 (47.33)		401 (68.46)	641 (53.86)	295 (45.67)	
Moderate	224 (37.28)	197 (35.71)	151 (22.96)		176 (26.05)	189 (33.65)	207 (36.12)		117 (23.31)	295 (34.09)	160 (36.10)	
Vigorous	88 (16.50)	61 (12.20)	51 (9.86)		52 (9.04)	67 (12.57)	81 (16.56)		35 (8.22)	93 (12.05)	72 (18.24)	
Smoking status, n (%)				< 0.001				< 0.001				< 0.001
Never smoker	323 (42.52)	353 (49.29)	306 (40.03)		330 (40.98)	331 (44.67)	321 (45.74)		240 (41.04)	497 (45.34)	245 (43.90)	
Former smoker	307 (47.69)	247 (35.29)	263 (37.40)		286 (36.58)	257 (39.55)	274 (44.20)		206 (36.12)	384 (38.31)	227 (47.15)	
Current smoker	73 (9.79)	103 (15.42)	134 (22.56)		148 (22.45)	87 (15.78)	75 (10.05)		107 (22.84)	148 (16.35)	55 (8.95)	
Alcohol consumption, n (%)				0.190				0.825				0.209
Non-drinker	542 (73.94)	558 (77.78)	570 (81.21)		601 (78.03)	546 (78.82)	523 (75.82)		445 (81.27)	820 (78.17)	405 (73.43)	
Mild to moderate	101 (16.62)	92 (15.86)	84 (11.70)		98 (14.50)	88 (14.39)	91 (15.51)		67 (11.90)	136 (15.22)	74 (16.42)	
Heavy	60 (9.43)	53 (6.36)	49 (7.09)		65 (7.47)	41 (6.79)	56 (8.67)		41 (6.83)	73 (6.62)	48 (10.15)	
CKM stage, n (%)				0.032				0.006				0.002
0	13 (3.55)	10 (2.73)	5 (1.09)		6 (1.13)	9 (2.13)	13 (3.94)		3 (0.54)	14 (2.34)	11 (4.28)	
1	25 (5.01)	21 (3.28)	15 (2.03)		17 (2.01)	19 (3.23)	25 (4.95)		11 (1.82)	30 (2.77)	20 (6.03)	
2	398 (61.33)	374 (56.61)	373 (58.23)		403 (56.84)	343 (54.76)	399 (63.90)		297 (58.17)	539 (56.44)	309 (63.27)	
3	145 (14.66)	161 (17.75)	148 (13.48)		166 (16.29)	159 (17.32)	129 (12.79)		115 (14.93)	237 (16.86)	102 (13.01)	
4	122 (15.45)	137 (19.63)	162 (25.18)		172 (23.73)	145 (22.55)	104 (14.42)		127 (24.55)	209 (21.59)	85 (13.41)	
Self-reported cancer, n (%)	110 (20.16)	96 (16.10)	91 (16.35)	0.155	102 (16.59)	97 (18.13)	98 (18.05)	0.749	74 (17.27)	143 (16.63)	80 (19.61)	0.378
Hemoglobin A1c, mean (SD), %	5.70 (0.04)	6.00 (0.07)	5.81 (0.05)	0.002	5.86 (0.07)	5.87 (0.06)	5.78 (0.06)	0.550	5.85 (0.08)	5.90 (0.06)	5.70 (0.05)	0.020
TC, mean (SD), mg/dL	210.42 (2.42)	213.71 (2.07)	214.70 (2.23)	0.232	213.91 (2.57)	213.07 (2.50)	211.77 (2.70)	0.782	214.28 (2.77)	213.49 (1.85)	210.64 (2.84)	0.479
HDL-C, mean (SD), mg/dL	52.36 (0.71)	51.44 (1.12)	52.93 (1.00)	0.491	51.88 (1.07)	52.38 (1.08)	52.39 (0.81)	0.862	51.76 (1.24)	52.30 (1.05)	52.47 (0.87)	0.902
eGFR, mean (SD), ml/min/1.73m^2^	78.51 (0.85)	77.36 (0.95)	74.55 (1.34)	0.062	74.34 (1.25)	77.38 (1.00)	78.52 (0.97)	0.013	73.11 (1.48)	77.56 (0.74)	78.64 (1.05)	0.006
CRP, mean (SD), mg/dl	0.52 (0.06)	0.50 (0.03)	0.59 (0.05)	0.191	0.57 (0.04)	0.52 (0.05)	0.53 (0.06)	0.636	0.60 (0.05)	0.54 (0.04)	0.49 (0.07)	0.467

Abbreviations: BMI, body mass index; CKM, cardiovascular-kidney-metabolic syndrome; CRP, C-reactive protein; DII, dietary inflammatory index; DOBS, dietary oxidative balance score; eGFR, estimated glomerular filtration rate; HDL-C, high-density lipoprotein cholesterol; HEI-2015, Healthy Eating Index-2015; SBP, systolic blood pressure; SD, standard deviation; TC, total cholesterol; UACR, urine albumin to creatinine ratio

### Associations between dietary indices and DNAmAAs

Table 2 presents the associations of DII, DOBS, and their composite index with DNAmAA measures. As a continuous variable, higher DII was significantly associated with increased odds of HorvathAA (OR = 1.23, 95% CI: 1.06–1.43), SkinBloodAA (OR = 1.15, 95% CI: 1.01–1.32), GrimAA (OR = 1.45, 95% CI: 1.28–1.62), and DunedinPoAm (OR = 1.05, 95% CI: 1.03–1.07), corresponding to a 45% higher odds of accelerated aging for GrimAA per unit increase in DII. Categorically, the highest DII tertile showed significantly elevated odds for HorvathAA (OR = 2.14, 95% CI: 1.15–3.97), GrimAA (OR = 3.63, 95% CI: 2.32–5.70), and DunedinPoAm (OR = 1.19, 95% CI: 1.08–1.28), with a significant increasing trend in odds across DII tertiles (all P for trend < 0.05). Higher DOBS as a continuous variable was associated with lower odds of GrimAA (OR = 0.92, 95% CI: 0.90–0.95) and DunedinPoAm (OR = 0.99, 95% CI: 0.98–0.99). Categorically, the highest DOBS tertile was associated with lower odds of GrimAA (OR = 0.38, 95% CI: 0.24–0.58) and DunedinPoAm (OR = 0.88, 95% CI: 0.80–0.95), with a significant decreasing trend across DOBS tertiles (all P for trend < 0.05). In combined analyses, an anti-inflammatory and antioxidant diet was associated with significantly reduced odds of GrimAA (OR = 0.27, 95% CI: 0.16–0.45) and DunedinPoAm (OR = 0.82, 95% CI: 0.74–0.90) compared with a pro-inflammatory and pro-oxidative diet, corresponding to approximately 73% and 18% lower odds, respectively.

**Table 2:** Associations between DII, DOBS, different combinations of DII and DOBS and DNAmAAs

	DNAmAA																			
	HorvathAA		HannumAA		SkinBloodAA		PhenoAA		GrimAA		ZhangAA		LinAA		WeiderAA		VidalBraloAA		DunedinPoAm	
	OR (95% CI)	*P*	OR (95% CI)	*P*	OR (95% CI)	*P*	OR (95% CI)	*P*	OR (95% CI)	*P*	OR (95% CI)	*P*	OR (95% CI)	*P*	OR (95% CI)	*P*	OR (95% CI)	*P*	OR (95% CI)	*P*
DII as continuous variable	1.23 (1.06–1.43)	0.010	1.12 (0.96–1.30)	0.160	1.15 (1.01–1.32)	0.050	1.19 (0.98–1.43)	0.080	1.45 (1.28–1.62)	< 0.001	1.02 (0.97–1.07)	0.400	1.08 (0.87–1.34)	0.490	0.97 (0.76–1.23)	0.810	1.01 (0.88–1.16)	0.860	1.05 (1.03–1.07)	< 0.001
DII as categorical variable																				
ertile 1 (< 0.87)	Reference		Reference		Reference		Reference		Reference		Reference		Reference		Reference		Reference		Reference	
Tertile 2 (0.87–2.38)	1.07 (1.95–3.60)	0.030	1.32 (0.72–2.44)	0.280	1.54 (0.89–2.66)	0.130	1.93 (0.91–4.10)	0.090	2.25 (1.45–3.49)	< 0.001	1.06 (0.87–1.30)	0.550	1.38 (0.58–3.29)	0.470	1.21 (0.47–3.16)	0.190	1.19 (0.68–2.05)	0.560	1.09 (1.01–1.20)	0.030
Tertile 3 (≥ 2.38)	2.14 (1.15–3.97)	0.020	1.21 (0.65–2.27)	0.540	1.32 (0.76–2.34)	0.330	1.49 (0.69–3.22)	0.300	3.63 (2.32–5.70)	< 0.001	1.01 (0.82–1.23)	0.950	1.11 (0.46–2.69)	0.820	1.17 (0.44–3.13)	0.740	1.09 (0.62–1.93)	0.760	1.19 (1.08–1.28)	< 0.001
P for trend	0.017		0.549		0.336		0.312		< 0.001		0.957		0.829		0.746		0.765		< 0.001	
DOBS as continuous variable	0.97 (0.93–1.01)	0.100	1.00 (0.96–1.04)	0.960	0.98 (0.95–1.02)	0.310	0.97 (0.92–1.01)	0.170	0.92 (0.90–0.95)	< 0.001	1.00 (0.99–1.01)	0.830	1.00 (0.95–1.06)	0.900	1.02 (0.96–1.08)	0.440	1.00 (0.97–1.04)	0.950	0.99 (0.98–0.99)	< 0.001
DOBS as categorical variable																				
Tertile 1 (< 10)	Reference		Reference		Reference		Reference		Reference		Reference		Reference		Reference		Reference		Reference	
Tertile 2 (10–17)	1.19 (0.65–2.14)	0.580	1.45 (0.79–2.61)	0.230	1.35 (0.79–2.34)	0.270	1.08 (0.52–2.27)	0.820	0.80 (0.52–1.25)	0.330	1.15 (0.94–1.40)	0.160	1.35 (0.58–3.16)	0.490	1.06 (0.41–2.69)	0.910	1.51 (0.87–2.59)	0.150	0.97 (0.89–1.05)	0.440
Tertile 3 (≥ 17)	0.73 (0.40–1.35)	0.320	0.87 (0.47–1.60)	0.650	2.34 (0.45–1.36)	0.380	0.63 (0.30–1.34)	0.230	0.38 (0.24–0.58)	< 0.001	1.00 (0.81–1.22)	0.960	1.01 (0.42–2.39)	0.990	1.12 (0.43–2.89)	0.820	0.98 (0.57–1.72)	0.960	0.88 (0.80–0.95)	0.002
P for trend	0.340		0.681		0.414		0.238		< 0.001		0.997		0.970		0.822		0.997		0.002	
Different combinations of DII and DOBS																				
Pro-inflammatory and pro-oxidative diet	Reference		Reference		Reference		Reference		Reference		Reference		Reference		Reference		Reference		Reference	
Composite diet category	0.91 (0.51–1.67)	0.770	1.16 (0.64–2.12)	0.610	1.20 (0.70–2.05)	0.520	0.88 (0.42–1.82)	0.720	0.57 (0.37–0.87)	0.01	1.07 (0.88–1.31)	0.510	1.42 (0.61–3.32)	0.420	1.58 (0.63–4.06)	0.330	1.20 (0.70–2.08)	0.510	0.90 (0.84–0.90)	0.020
Anti-inflammatory and antioxidant diet	0.59 (0.29–1.19)	0.140	0.83 (0.41–1.67)	0.590	0.73 (0.39–1.38)	0.330	0.55 (0.23–1.32)	0.190	0.27 (0.16–0.45)	< 0.001	0.99 (0.78–1.25)	0.920	1.06 (0.39–2.89)	0.910	1.35 (0.44–4.06)	0.600	0.94 (0.49–1.79)	0.850	0.82 (0.74–0.90)	< 0.001

Model 1 was adjusted for age, sex, and race and ethnicity

Model 2 were adjusted for age, sex, race and ethnicity, poverty income ratio, smoking status, and alcohol consumption

Model 3 were adjusted for age, sex, race and ethnicity, poverty income ratio, smoking status, and alcohol consumption, physical activity, Healthy Eating Index-2015, and C-reactive protein

Abbreviations: CKM, cardiovascular-kidney-metabolic syndrome; CI, confidence interval; DII, dietary inflammatory index; DOBS, dietary oxidative balance score; OR, odds ratio

### Associations between dietary indices and CKM stages

Table 3 shows the relationships between the DII, DOBS, and their composite index with CKM stages. When treated as a continuous variable, higher DII scores were significantly linked to greater odds of progressing to advanced CKM stages in all models, with an odds ratio (OR) of 1.24 (95% CI: 1.15–1.33) in the fully adjusted model. In categorical analyses, a significant positive trend was observed, with the highest DII tertile showing substantially elevated odds (fully adjusted model: OR = 1.97; 95% CI: 1.38–2.80). In contrast, higher DOBS scores as a continuous variable were linked to lower odds of CKM stage progression, with an odds ratio (OR) of 0.96 (95% CI: 0.94–0.98) in the fully adjusted model. The highest DOBS tertile also showed a protective effect (fully adjusted model: OR = 0.62; 95% CI: 0.44–0.89). Additionally, compared to a pro-inflammatory and pro-oxidative diet, an anti-inflammatory and antioxidant diet was significantly associated with reduced odds of advancing to higher CKM stages (fully adjusted model: OR = 0.53; 95% CI: 0.36–0.80).

**Table 3:** Associations between DII, DOBS, different combinations of DII and DOBS with CKM stages

	CKM stage					
	Model 1		Model 2		Model 3	
	OR (95% CI)	*P*	OR (95% CI)	*P*	OR (95% CI)	*P*
Continuous DII	1.21 (1.14–1.28)	< 0.001	1.18 (1.11–1.25)	< 0.001	1.24 (1.15–1.33)	< 0.001
Categorical DII						
Tertile 1 (< 0.87)	*Reference*		*Reference*		*Reference*	
Tertile 2 (0.87–2.38)	1.52 (1.24–1.87)	< 0.001	1.43 (1.16–1.76)	0.003	1.52 (1.21–1.90)	0.002
Tertile 3 (≥ 2.38)	2.04 (1.51–2.75)	< 0.001	1.78 (1.32–2.40)	0.001	1.97 (1.38–2.80)	0.001
P for trend		< 0.001		0.001		0.001
Continuous DOBS	0.96 (0.94–0.98)	< 0.001	0.97 (0.95–0.98)	0.001	0.96 (0.94–0.98)	0.002
Categorical DOBS						
Tertile 1 (< 10)	*Reference*		*Reference*		*Reference*	
Tertile 2 (10–17)	0.86 (0.70–1.27)	0.693	1.02 (0.77–1.35)	0.902	1.02 (0.75–1.38)	0.920
Tertile 3 (≥ 17)	0.85 (0.42–0.80)	0.003	0.64 (0.47–0.87)	0.010	0.62 (0.44–0.89)	0.017
P for trend		0.002		0.007		0.013
Different combinations of DII and DOBS						
Pro-inflammatory and pro-oxidative diet	*Reference*		*Reference*		*Reference*	
Composite diet category	0.89 (0.65–1.21)	0.452	1.02 (0.77–1.37)	0.873	0.95 (0.69–1.30)	0.756
Anti-inflammatory and antioxidant diet	0.49 (0.34–0.70)	< 0.001	0.57 (0.40–0.81)	0.005	0.53 (0.36–0.80)	0.007

Model 1 was adjusted for age sex, and race and ethnicity

Model 2 were adjusted for age, sex, race and ethnicity, poverty income ratio, smoking status, and alcohol consumption

Model 3 were adjusted for age, sex, race and ethnicity, poverty income ratio, smoking status, and alcohol consumption, physical activity, Healthy Eating Index-2015, and C-reactive protein

Abbreviations: CKM, cardiovascular-kidney-metabolic syndrome; CI, confidence interval; DII, dietary inflammatory index; DOBS, dietary oxidative balance score; OR, odds ratio

### Associations between dietary indices and mortality in participants with CKM syndrome

Table 4 displays the relationships between dietary indices and mortality among participants with CKM syndrome. Higher DII scores were significantly associated with increased risks with all-cause (HR = 1.08, 95% CI: 1.03–1.13) and cardiovascular mortality (HR = 1.17, 95% CI: 1.07–1.28). In tertile analyses, participants in the highest DII tertile had higher risks of all-cause (HR = 1.34, 95% CI: 1.13–1.59), cardiovascular (HR = 1.71, 95% CI: 1.23–2.39), and non-cardiovascular mortality (HR = 1.24, 95% CI: 1.01–1.51) compared with those in the lowest tertile. In contrast, higher DOBS scores were inversely associated with all-cause (HR = 0.98, 95% CI: 0.97–0.99), cardiovascular (HR = 0.96, 95% CI: 0.94–0.98), and non-cardiovascular mortality (HR = 0.99, 95% CI: 0.98-1.00). The highest DOBS tertile showed substantially reduced risks for all-cause (HR = 0.77, 95% CI: 0.66–0.90), and cardiovascular mortality (HR = 0.61, 95% CI: 0.45–0.84). Moreover, participants following an anti-inflammatory and antioxidant dietary pattern had significantly lower mortality risks than those with a pro-inflammatory and pro-oxidative dietary pattern, with a 30% reduction in all-cause (95% CI: 0.58–0.84), 40% reduction in cardiovascular (95% CI: 0.42–0.87), and 26% reduction in non-cardiovascular mortality (95% CI: 0.59–0.92). The consistency of the results was robust in sensitivity analyses that excluded participants who died within 2 years or had a cancer history (eTable 8 & eTable 9).

**Table 4:** Associations between DII, DOBS, different combinations of DII and DOBS with mortality in population with CKM syndrome

	All-cause mortality		Cardiovascular mortality		Non-cardiovascular mortality	
	HR (95% CI)	*P*	HR (95% CI)	*P*	HR (95% CI)	*P*
Continuous DII	1.08 (1.03–1.13)	< 0.001	1.17 (1.07–1.28)	< 0.001	1.05 (1.00-1.11)	0.055
Categorical DII						
Tertile 1 (< 0.87)	*Reference*		*Reference*		*Reference*	
Tertile 2 (0.87–2.38)	1.11 (0.95–1.30)	0.176	1.35 (0.99–1.83)	0.056	1.04 (0.87–1.25)	0.661
Tertile 3 (≥ 2.38)	1.34 (1.13–1.59)	< 0.001	1.71 (1.23–2.39)	0.002	1.24 (1.01–1.51)	0.036
P for trend		< 0.001		0.001		0.032
Continuous DOBS	0.98 (0.97, 0.99)	< 0.001	0.96 (0.94, 0.98)	< 0.001	0.99 (0.98, 1.00)	0.029
Categorical DOBS						
Tertile 1 (< 10)	*Reference*		*Reference*		*Reference*	
Tertile 2 (10–17)	0.90 (0.78–1.03)	0.125	0.75 (0.58–0.99)	0.043	0.96 (0.81–1.13)	0.595
Tertile 3 (≥ 17)	0.77 (0.66–0.90)	0.001	0.61 (0.45–0.84)	0.002	0.84 (0.69–1.01)	0.060
P for trend		0.001		0.002		0.065
Different combinations of DII and DOBS						
Pro-inflammatory and pro-oxidative diet	*Reference*		*Reference*		*Reference*	
Composite diet category	0.80 (0.69–0.92)	0.001	0.72 (0.55–0.94)	0.017	0.82 (0.70–0.97)	0.021
Anti-inflammatory and antioxidant diet	0.70 (0.58–0.84)	< 0.001	0.60 (0.42–0.87)	0.006	0.74 (0.59–0.92)	0.006
P for trend		< 0.001		0.005		0.005

All models were adjusted for age, sex, race and ethnicity, poverty income ratio, smoking status, and alcohol consumption, physical activity, Healthy Eating Index-2015, C-reactive protein, and self-reported cancer

Abbreviations: CKM, cardiovascular-kidney-metabolic syndrome; CI, confidence interval; DII, dietary inflammatory index; DOBS, dietary oxidative balance score; OR, odds ratio

### GrimAA and DunedinPoAm mediate the association between dietary indices and CKM stages

**Fig. 1:**
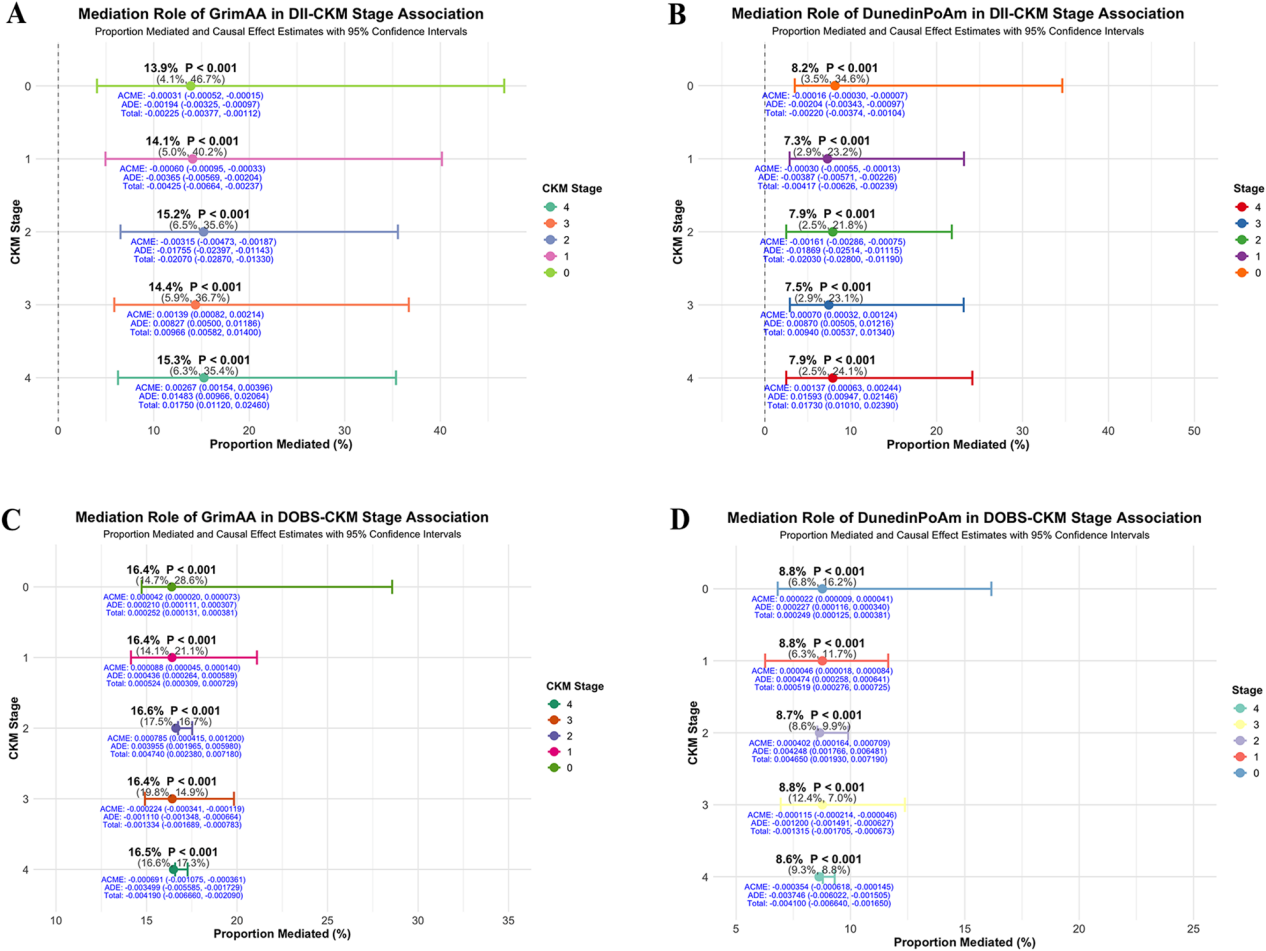
Mediation role of GrimAA and DunedinPoAm in the association between dietary indices and CKM stages. **A**: Mediation role of GrimAA in DII-CKM stage association; **B**: Mediation role of DunedinPoAm in DII-CKM stage association; **C**: Mediation role of GrimAA in DOBS-CKM stage association; **D**: Mediation role of DunedinPoAm in DOBS-CKM stage association. Data are presented as proportion mediated (%) with 95% confidence intervals and corresponding causal effect estimates (ACME: average causal mediation effect; Total: total effect). All *P* < 0.001. ACME, average causal mediation effect; CKM, cardiovascular-kidney-metabolic; DII, Dietary Inflammatory Index; DOBS = Dietary Oxidative Balance Score; DunedinPoAm, Dunedin pace of aging; GrimAA, GrimAge2Mort acceleration

Mediation analysis indicated that both GrimAA and DunedinPoAm significantly mediated the relationships between dietary indices and CKM stages (Fig. 1). Specifically, for the association of higher DII with advanced CKM stages: GrimAA mediated 13.9%-16.5% (95% CI range: 4.1%-46.7%, all *P* < 0.001) of the relationship, with corresponding average causal mediation effects (ACME) ranging from − 0.00060 to 0.00267; DunedinPoAm mediated 7.3%-8.8% (95% CI range: 2.5%-34.6%, all *P* < 0.001) of this association. Conversely, in the protective pathway of DOBS for CKM stage progression: GrimAA accounted for 16.4%-16.6% (95% CI range: 6.3%-17.3%, all *P* < 0.001) of the risk reduction, with ACME ranging from 0.000088 to 0.000224; DunedinPoAm explained 7.5%-8.8% (95% CI range: 2.9%-24.1%, all *P* < 0.001) of the protective effect conferred by DOBS.

### GrimAA and DunedinPoAm mediate the association between dietary indices and mortality in CKM syndrome

**Fig. 2:**
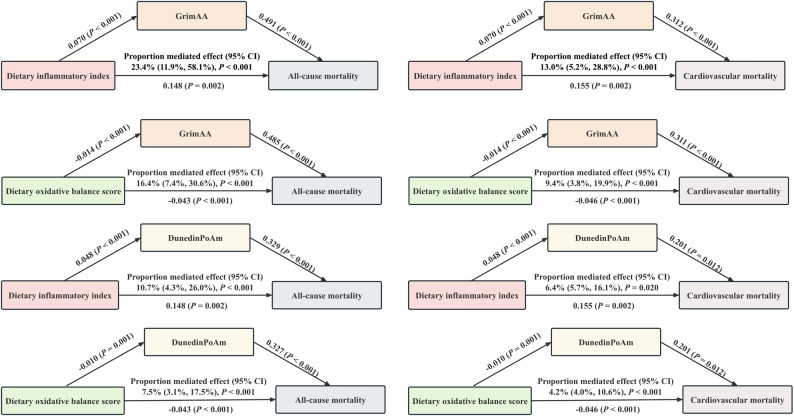
Mediation role of GrimAA and DunedinPoAm in the association between dietary indices and mortality in CKM syndrome. Data are presented as proportion mediated (%) with 95% confidence intervals and corresponding effect estimates. CKM, cardiovascular-kidney-metabolic; DunedinPoAm, Dunedin pace of aging; GrimAA, GrimAge2Mort acceleration

Mediation analysis revealed that GrimAA and DunedinPoAm significantly mediated the associations between dietary indices and mortality (Fig. 2). Specifically, for DII-related effects: GrimAA accounted for 23.4% (95% CI: 11.9%-58.1%, *P* < 0.001) of the effect on all-cause mortality and 13.0% (95% CI: 5.2%-28.8%, *P* < 0.001) of its effect on cardiovascular mortality; DunedinPoAm mediated 10.7% (95% CI: 4.3%-26.0%, *P* < 0.001) of the effect of DII on all-cause mortality and 6.4% (95% CI: 5.7%-16.1%, *P* = 0.020) of its effect on cardiovascular mortality. In contrast, for DOBS-related protective effects: GrimAA mediated 16.4% (95% CI: 7.4%-30.6%, *P* < 0.001) of the reduction in all-cause mortality and 9.4% (95% CI: 3.8%-19.9%, *P* < 0.001) of the reduction in cardiovascular mortality; DunedinPoAm explained 7.5% (95% CI: 3.1%-17.5%, *P* < 0.001) of the protective effect of DOBS on all-cause mortality and 4.2% (95% CI: 4.0%-10.6%, *P* < 0.001) of its protective effect on cardiovascular mortality.

## Discussion

In this comprehensive, nationally representative study of U.S. adults with CKM syndrome, we observed a consistent association between dietary inflammatory and oxidative potential, epigenetic aging measures, and mortality outcomes. Our findings indicate that a pro-inflammatory and pro-oxidative diet, characterized by a high DII and a low DOBS, is significantly associated with accelerated epigenetic aging, particularly as measured by GrimAA and DunedinPoAm. Specifically, a high DII score correlates with more advanced stages of CKM and increased risks of all-cause, cardiovascular, and non-cardiovascular mortality, whereas antioxidant-rich dietary patterns were associated with lower risks. Additionally, mediation analyses suggest that GrimAA and DunedinPoAm may partially account for the observed associations, accounting for a substantial portion of the relationship between dietary inflammatory and oxidative potential and mortality.

In the present study, higher DII scores were associated with increased odds of accelerated epigenetic aging across multiple clocks, whereas higher DOBS values were associated with lower odds, particularly for GrimAA and DunedinPoAm. Accumulating evidence indicates that high-quality dietary patterns, characterized by unprocessed foods and anti-inflammatory properties, are associated with a significantly slower pace of epigenetic aging [35–38]. A study conducted by Bordoni et al. with 760 adults found that high diet quality, characterized by reduced processed food intake, lower glycemic load, and adequate vitamin consumption, is related to decelerated biological aging measured by Horvath, Hannum, and Levine [37]. Overall, our findings extend this evidence to CKM syndrome and suggest that dietary inflammatory and oxidative potential may play an important role in shaping biological aging trajectories. Our present study extends this evidence to CKM syndrome, suggesting that higher DII scores are associated with accelerated epigenetic aging across multiple clocks including HorvathAA, SkinBloodAA, GrimAA, and DunedinPoAm, in contrast, a high DOBS was associated with notably lower odds of accelerated aging as measured by GrimAA and DunedinPoAm.

The relationship between dietary patterns and disease incidence and mortality has been well-established. Substantial epidemiological evidence indicates that pro-inflammatory diets are associated with elevated risks of CVD, metabolic disorders, autoinflammatory conditions, cancer, and cognitive decline [5–8]. Prospective cohort studies further demonstrate that higher DII scores are independently linked to the development of type 2 diabetes, non-alcoholic fatty liver disease, CKD, and atherosclerotic cardiovascular events [10–12, 39]. For example, a pooled analysis of three prospective cohorts revealed that higher empirical dietary inflammatory pattern scores were associated with increased CVD risk [11]. Similarly, a UK Biobank study involving 106,870 participants found that higher DII scores were strongly linked to an increased risk of CKD, while following healthy dietary patterns was associated with a reduced risk [12]. Another study combining data from the UK Biobank and NHANES demonstrated that both greater dietary diversity and lower inflammatory potential were independently associated with reduced risks of all-cause mortality and the incidence of type 2 diabetes [39]. Accumulating evidence further supports the association between higher DII scores and increased mortality [40, 41]. An analysis of two Spanish cohorts and a meta-analysis of 12 prospective studies by Garcia-Arellano et al. confirmed elevated DII scores were significantly associated with higher all-cause mortality. Consistently, a Mediterranean population study reported a 13% increase in all-cause mortality risk per 1-SD increase in DII [40, 41].

Our study extends this evidence to CKM syndrome, demonstrating that higher DII scores are significantly associated with more advanced CKM stages, while higher DOBS exhibits protective effects. Beyond disease progression, we further found a dose-response relationship between dietary patterns and mortality outcomes. Specifically, pro-inflammatory dietary patterns were associated with higher risks of all-cause and cardiovascular mortality among CKM patients, whereas antioxidant-rich dietary patterns were associated with lower risks. This relationship was supported by consistent trends across increasing levels of DII and DOBS, with progressively higher mortality risks observed across DII categories and lower risks across DOBS categories, indicating a graded association rather than a simple group comparison. The consistency of these findings across both disease progression and mortality endpoints underscores the importance of evaluating overall dietary quality rather than focusing on isolated nutrients in CKM syndrome management. This comprehensive approach to nutritional assessment better reflects the complex interplay between dietary components and their collective impact on disease pathogenesis. The demonstrated benefits of anti-inflammatory and antioxidant diets support the integration of dietary pattern evaluation as a fundamental component in the clinical management of CKM syndrome.

Recent studies have further highlighted the role of dietary inflammatory and oxidative indices in CKM-related outcomes. For example, emerging evidence has demonstrated that higher DII and lower DOBS are associated with increased risk of CKM progression and adverse cardiovascular outcomes in large population-based cohorts [42–44]. Our findings are broadly consistent with these observations. Importantly, beyond confirming these associations, the present study further integrates epigenetic aging markers, suggesting that biological aging may represent an intermediate pathway linking dietary patterns with CKM progression and mortality.

While the associations between dietary patterns, age-related diseases, and mortality are well-documented, the underlying biological mechanisms have remained incompletely understood. Our mediation analyses suggest that part of the association between dietary inflammatory and oxidative potential and CKM progression or mortality may operate through differences in biological aging as captured by epigenetic clocks, particularly GrimAA and DunedinPoAm. For CKM progression, GrimAA mediates 13.9%-16.5% of the adverse association between higher DII and advanced CKM stages and 16.4%-16.6% of the protective effect of higher DOBS, while DunedinPoAm accounts for 7.3%-8.8% of the DII-related risk and 7.5%-8.8% of the DOBS-related benefit. For mortality in CKM syndrome, GrimAA explains 23.4% of the DII-induced all-cause mortality risk and 13.0% of cardiovascular mortality risk, while mediating 16.4% of the DOBS-related all-cause mortality reduction and 9.4% of cardiovascular mortality reduction. DunedinPoAm explains 10.7% of DII’s all-cause mortality effect and 6.4% to its cardiovascular mortality effect, with 7.5% and 4.2% mediation of DOBS’s protective effect on all-cause and cardiovascular mortality, respectively. These findings are consistent with the hypothesis that dietary patterns, biological aging, and CKM outcomes may be interconnected, indicating that diet contributes to CKM progression and elevated mortality risk, at least in part, by accelerating biological aging. As quantifiable measures of biological age, epigenetic clocks reflect the cumulative effect of long-term dietary exposures on physiological function, thereby suggesting a potential biological link between dietary patterns and clinical outcomes. This insight resonates with the conceptual framework advanced by López-Otín et al., which identifies dysregulated nutrient sensing and metabolism as core hallmarks of aging [45]. Our results empirically anchor dietary patterns within this framework, positing that clinical manifestations of CKM progression represent, in part, downstream effects of diet-induced increases in biological age. Consequently, GrimAA and DunedinPoAm may reflect cumulative physiological changes associated with long-term dietary exposures.

Notably, GrimAA consistently accounted for a larger proportion of the observed associations between dietary indices and mortality compared with DunedinPoAm. This pattern may reflect the stronger sensitivity of GrimAge to inflammation- and mortality-related biological pathways, as it incorporates DNA methylation surrogates of plasma proteins and smoking pack-years, which are closely linked to cardiometabolic risk. In contrast, DunedinPoAm captures the overall pace of systemic aging across multiple organ systems and may therefore reflect a broader, but less diet-specific, aging signal. The comparatively smaller mediation proportions observed for DunedinPoAm suggest that while dietary exposures may contribute to global physiological aging, their effects may be more strongly concentrated in inflammation-driven and mortality-related pathways, which are more directly captured by GrimAA.

Although dietary intake in this study was assessed using a single 24-hour recall, the inflammatory and oxidative potential of the diet summarized by DII and DOBS may still reflect broader dietary tendencies at the population level. Previous epidemiological studies have shown that nutrient-based indices derived from a single recall can capture meaningful variation in dietary patterns and are widely used in NHANES-based research. Therefore, while a single-day dietary snapshot cannot fully represent habitual intake, it may still provide informative estimates of the overall inflammatory and oxidative characteristics of the diet in large population studies.

The observed associations may be consistent with several biological processes operating at molecular, cellular, and systemic levels. Pro-inflammatory and pro-oxidative diets may contribute to biological processes linked to accelerated epigenetic aging by establishing a state of chronic systemic inflammation and elevated oxidative stress. This pro-inflammatory milieu promotes cellular senescence through multiple pathways, including NF-κB activation and reactive oxygen species generation, while simultaneously altering DNA methyltransferase activity and fidelity, leading to widespread epigenetic dysregulation [46–48]. The GrimAge clock shows particular sensitivity to these processes, as it incorporates methylation sites in genes encoding inflammation-related proteins and tissue integrity factors, effectively capturing the molecular footprint of diet-induced inflammatory burden [49]. Concurrently, the DunedinPoAm measure, reflecting the pace of aging across multiple organ systems, captures the cumulative burden of metabolic stress and molecular damage resulting from long-term adverse dietary exposures [50, 51]. This persistent diet-induced physiological stress progressively dysregulates cardiovascular, renal, and metabolic systems, which are central to CKM syndrome. Thus, the methylation profiles derived from these epigenetic clocks represent the biological embedding of dietary exposures, providing a mechanistic explanation for their association with CKM progression and mortality.

A key strength of this study is its novel integration of comprehensive dietary data, a panel of epigenetic clocks, and long-term mortality follow-up within a representative cohort, which facilitates the exploration of complex mediating pathways. The use of multiple epigenetic clocks offers a more nuanced perspective on the aging process, while rigorous adjustments for confounders, including overall diet quality as measured by the HEI-2015, strengthen the inference regarding the specific roles of inflammatory and oxidative dietary components. At the same time, the relationship between HEI-2015 and the DII/DOBS warrants careful consideration. As a guideline-based measure of overall dietary quality, the HEI inherently reflects patterns of food intake that may also influence the inflammatory and oxidative characteristics of the diet [52]. Thus, diets with higher HEI scores may naturally overlap with more anti-inflammatory and antioxidant dietary profiles. In this context, DII and DOBS may be viewed not only as complementary dietary indices, but also as potential biological dimensions through which overall dietary quality becomes linked to CKM progression and mortality. This conceptual overlap does not negate their relevance, but it does suggest that the independent effects of these indices should be interpreted with caution. Future studies should further disentangle the joint and distinct contributions of overall dietary guideline adherence, dietary inflammatory potential, and oxidative balance, particularly in the context of evolving U.S. dietary recommendations.

However, several limitations warrant careful consideration. The observational design of our study limits the ability to draw definitive causal conclusions. In particular, mediation analysis in observational data cannot establish causal pathways, and the identified mediating proportions should be interpreted as statistical decompositions of associations rather than definitive biological mediation. Although we adjusted for key confounders and conducted sensitivity analyses to address potential reverse causality, residual confounding from unmeasured variables such as psychosocial stress or environmental exposures remains possible. Second, dietary intake was assessed using a single 24-hour dietary recall, which may not fully capture habitual dietary patterns relevant to long-term biological processes such as epigenetic aging. Although this approach is commonly used in large epidemiological studies including NHANES, day-to-day variation in diet may introduce measurement error. Such non-differential misclassification would likely bias the observed associations toward the null rather than generate spurious positive findings. Third, DNA methylation was assessed only at baseline, which does not account for potential changes in epigenetic status that may occur over the course of the follow-up period. Furthermore, certain lifestyle factors including physical activity, sleep quality, and socioeconomic status that influence biological aging were not fully accounted for in our analyses. Finally, while NHANES provides excellent generalizability to the U.S. population, the applicability of our findings to other ethnicities and healthcare settings requires external validation.

## Conclusions

In this nationally representative study of U.S. adults, we demonstrate that pro-inflammatory and pro-oxidative diets are significantly associated with accelerated epigenetic aging, as measured by GrimAA and DunedinPoAm, and with adverse clinical outcomes including CKM progression and increased mortality. Conversely, anti-inflammatory and antioxidant diets were associated with protective effects, slowing biological aging and reducing the risk of CKM progression and mortality. Mediation analyses further reveal that epigenetic age acceleration, particularly through GrimAA and DunedinPoAm, significantly mediates the pathway linking dietary patterns to CKM outcomes. These findings underscore the importance of dietary quality in modulating biological aging and suggest that interventions aimed at reducing dietary inflammatory and oxidative potential may mitigate the burden of CKM syndrome. Future research should validate these findings in varied populations and investigate the potential of epigenetic clocks as biomarkers in dietary intervention studies.

## Electronic supplementary material

Below is the link to the electronic supplementary material.

Supplementary Material 1

## Data Availability

The data supporting the findings of this study are publicly available from the NHANES. Access can be requested through the official NHANES website: https://www.cdc.gov/nchs/nhanes/index.htm.

## References

[1] Ndumele CE, Neeland IJ, Tuttle KR, Chow SL, Mathew RO, Khan SS, Coresh J, Baker-Smith CM, Carnethon MR, Després JP, et al. A Synopsis of the Evidence for the Science and Clinical Management of Cardiovascular-Kidney-Metabolic (CKM) Syndrome: A Scientific Statement From the American Heart Association. Circulation. 2023;148:1636–64. 10.1161/cir.0000000000001186. 37807920 10.1161/CIR.0000000000001186

[2] Ndumele CE, Rangaswami J, Chow SL, Neeland IJ, Tuttle KR, Khan SS, Coresh J, Mathew RO, Baker-Smith CM, Carnethon MR, et al. Cardiovascular-Kidney-Metabolic Health: A Presidential Advisory From the American Heart Association. Circulation. 2023;148:1606–35. 10.1161/cir.0000000000001184. 37807924 10.1161/CIR.0000000000001184

[3] Aggarwal R, Ostrominski JW, Vaduganathan M. Prevalence of Cardiovascular-Kidney-Metabolic Syndrome Stages in US Adults, 2011–2020. JAMA. 2024;331:1858–60. 10.1001/jama.2024.6892. 38717747 10.1001/jama.2024.6892 PMC11079779

[4] Li N, Li Y, Cui L, Shu R, Song H, Wang J, Chen S, Liu B, Shi H, Gao H, et al. Association between different stages of cardiovascular-kidney-metabolic syndrome and the risk of all-cause mortality. Atherosclerosis. 2024;397:118585. 10.1016/j.atherosclerosis.2024.118585. 39255681 10.1016/j.atherosclerosis.2024.118585

[5] Farazi M, Jayedi A, Shab-Bidar S. Dietary inflammatory index and the risk of non-communicable chronic disease and mortality: an umbrella review of meta-analyses of observational studies. Crit Rev Food Sci Nutr. 2023;63:57–66. 10.1080/10408398.2021.1943646. 34176394 10.1080/10408398.2021.1943646

[6] Yu X, Pu H, Voss M. Overview of anti-inflammatory diets and their promising effects on non-communicable diseases. Br J Nutr. 2024;132:898–918. 10.1017/s0007114524001405. 39411832 10.1017/S0007114524001405 PMC11576095

[7] Wang W, Liu Y, Li Y, Luo B, Lin Z, Chen K, Liu Y. Dietary patterns and cardiometabolic health: Clinical evidence and mechanism. MedComm. 2023;4:e212. 10.1002/mco2.212. 36776765 10.1002/mco2.212 PMC9899878

[8] Marx W, Veronese N, Kelly JT, Smith L, Hockey M, Collins S, Trakman GL, Hoare E, Teasdale SB, Wade A, et al. The Dietary Inflammatory Index and Human Health: An Umbrella Review of Meta-Analyses of Observational Studies. Adv Nutr (Bethesda Md). 2021;12:1681–90. 10.1093/advances/nmab037. 10.1093/advances/nmab037 PMC8483957 33873204

[9] Hernández-Ruiz Á, García-Villanova B, Guerra-Hernández EJ, Carrión-García CJ, Amiano P, Sánchez MJ, Molina-Montes E. Oxidative Balance Scores (OBSs) Integrating Nutrient, Food and Lifestyle Dimensions: Development of the NutrientL-OBS and FoodL-OBS. Antioxid (Basel Switzerland). 2022;11. 10.3390/antiox11020300. 10.3390/antiox11020300 PMC8868253 35204183

[10] Hariharan R, Odjidja EN, Scott D, Shivappa N, Hébert JR, Hodge A, de Courten B. The dietary inflammatory index, obesity, type 2 diabetes, and cardiovascular risk factors and diseases. Obes reviews: official J Int Association Study Obes. 2022;23:e13349. 10.1111/obr.13349. 10.1111/obr.13349 34708499

[11] Li J, Lee DH, Hu J, Tabung FK, Li Y, Bhupathiraju SN, Rimm EB, Rexrode KM, Manson JE, Willett WC, et al. Dietary Inflammatory Potential and Risk of Cardiovascular Disease Among Men and Women in the U.S. J Am Coll Cardiol. 2020;76:2181–93. 10.1016/j.jacc.2020.09.535. 33153576 10.1016/j.jacc.2020.09.535 PMC7745775

[12] Maroto-Rodriguez J, Ortolá R, Cabanas-Sanchez V, Martinez-Gomez D, Rodriguez-Artalejo F, Sotos-Prieto M. Diet quality patterns and chronic kidney disease incidence: a UK Biobank cohort study. Am J Clin Nutr. 2025;121:445–53. 10.1016/j.ajcnut.2024.12.005. 39667719 10.1016/j.ajcnut.2024.12.005

[13] Horvath S, Raj K. DNA methylation-based biomarkers and the epigenetic clock theory of ageing. Nat Rev Genet. 2018;19:371–84. 10.1038/s41576-018-0004-3. 29643443 10.1038/s41576-018-0004-3

[14] Noroozi R, Ghafouri-Fard S, Pisarek A, Rudnicka J, Spólnicka M, Branicki W, Taheri M, Pośpiech E. DNA methylation-based age clocks: From age prediction to age reversion. Ageing Res Rev. 2021;68:101314. 10.1016/j.arr.2021.101314. 33684551 10.1016/j.arr.2021.101314

[15] Duan R, Fu Q, Sun Y, Li Q. Epigenetic clock: A promising biomarker and practical tool in aging. Ageing Res Rev. 2022;81:101743. 10.1016/j.arr.2022.101743. 36206857 10.1016/j.arr.2022.101743

[16] Shan J, Tay JH, Ye KX, Guo J, Cao L, Zeng Y, Lee TS, Heok KE, Kennedy BK, Maier AB, Feng L. Lifestyle factors and DNA methylation-based aging clocks: cross-sectional and longitudinal associations in the Singapore diet and healthy aging cohort. J Prev Alzheimers Dis. 2026;13:100522. 10.1016/j.tjpad.2026.100522. 41763011 10.1016/j.tjpad.2026.100522 PMC12964021

[17] Szarc vel Szic K, Declerck K, Vidaković M, Vanden Berghe W. From inflammaging to healthy aging by dietary lifestyle choices: is epigenetics the key to personalized nutrition? Clin Epigenetics. 2015;7:33. 10.1186/s13148-015-0068-2. 25861393 10.1186/s13148-015-0068-2 PMC4389409

[18] Bell CG, Lowe R, Adams PD, Baccarelli AA, Beck S, Bell JT, Christensen BC, Gladyshev VN, Heijmans BT, Horvath S, et al. DNA methylation aging clocks: challenges and recommendations. Genome Biol. 2019;20:249. 10.1186/s13059-019-1824-y. 31767039 10.1186/s13059-019-1824-y PMC6876109

[19] Soto-Palma C, Niedernhofer LJ, Faulk CD, Dong X, Epigenetics. DNA damage, and aging. J Clin Investig. 2022;132. 10.1172/jci158446. 10.1172/JCI158446 PMC9374376 35968782

[20] Seale K, Horvath S, Teschendorff A, Eynon N, Voisin S. Making sense of the ageing methylome. Nat Rev Genet. 2022;23:585–605. 10.1038/s41576-022-00477-6. 35501397 10.1038/s41576-022-00477-6

[21] Tamman AJF, Koller D, Nagamatsu S, Cabrera-Mendoza B, Abdallah C, Krystal JH, Gelernter J, Montalvo-Ortiz JL, Polimanti R, Pietrzak RH. Psychosocial moderators of polygenic risk scores of inflammatory biomarkers in relation to GrimAge. Neuropsychopharmacology. 2024;49:699–708. 10.1038/s41386-023-01747-5. 37848731 10.1038/s41386-023-01747-5 PMC10876568

[22] Alimohammadi M, Makaremi S, Rahimi A, Asghariazar V, Taghadosi M, Safarzadeh E. DNA methylation changes and inflammaging in aging-associated diseases. Epigenomics. 2022;14:965–86. 10.2217/epi-2022-0143. 36043685 10.2217/epi-2022-0143

[23] Jiang M, Tian S, Liu S, Wang Y, Guo X, Huang T, Lin X, Belsky DW, Baccarelli AA, Gao X. Accelerated biological aging elevates the risk of cardiometabolic multimorbidity and mortality. Nat Cardiovasc Res. 2024;3:332–42. 10.1038/s44161-024-00438-8. 39196113 10.1038/s44161-024-00438-8 PMC13265155

[24] Wu S, Zhu J, Lyu S, Wang J, Shao X, Zhang H, Zhong Z, Liu H, Zheng L, Chen Y. Impact of DNA-Methylation Age Acceleration on Long-Term Mortality Among US Adults With Cardiovascular-Kidney-Metabolic Syndrome. J Am Heart Assoc. 2025;14:e039751. 10.1161/jaha.124.039751. 40118808 10.1161/JAHA.124.039751 PMC12132836

[25] Chen Y, Wu S, Liu H, Zhong Z, Bucci T, Wang Y, Zhao M, Liu Y, Yang Z, Gue Y, et al. Role of oxidative balance score in staging and mortality risk of cardiovascular-kidney-metabolic syndrome: Insights from traditional and machine learning approaches. Redox Biol. 2025;81:103588. 10.1016/j.redox.2025.103588. 40073760 10.1016/j.redox.2025.103588 PMC11950999

[26] Li J, Lei L, Wang W, Ding W, Yu Y, Pu B, Peng Y, Li Y, Zhang L, Guo Y. Social Risk Profile and Cardiovascular-Kidney-Metabolic Syndrome in US Adults. J Am Heart Association. 2024;13:e034996. 10.1161/jaha.124.034996. 10.1161/JAHA.124.034996 PMC11963957 39136302

[27] Minhas AMK, Mathew RO, Sperling LS, Nambi V, Virani SS, Navaneethan SD, Shapiro MD, Abramov D. Prevalence of the Cardiovascular-Kidney-Metabolic Syndrome in the United States. J Am Coll Cardiol. 2024;83:1824–6. 10.1016/j.jacc.2024.03.368. 38583160 10.1016/j.jacc.2024.03.368

[28] Zhong M, Liu CN, Chen Y. The Associations of Anthropometric Indices With Stages and Mortality in Cardiovascular-Kidney-Metabolic Syndrome: Insights From NHANES. Rev Cardiovasc Med. 2026;27:46650. 10.31083/rcm46650. 41789343 10.31083/RCM46650 PMC12960012

[29] Khan SS, Coresh J, Pencina MJ, Ndumele CE, Rangaswami J, Chow SL, Palaniappan LP, Sperling LS, Virani SS, Ho JE, et al. Novel Prediction Equations for Absolute Risk Assessment of Total Cardiovascular Disease Incorporating Cardiovascular-Kidney-Metabolic Health: A Scientific Statement From the American Heart Association. Circulation. 2023;148:1982–2004. 10.1161/cir.0000000000001191. 37947094 10.1161/CIR.0000000000001191

[30] Levey AS, de Jong PE, Coresh J, El Nahas M, Astor BC, Matsushita K, Gansevoort RT, Kasiske BL, Eckardt KU. The definition, classification, and prognosis of chronic kidney disease: a KDIGO Controversies Conference report. Kidney Int. 2011;80:17–28. 10.1038/ki.2010.483. 21150873 10.1038/ki.2010.483

[31] Shivappa N, Steck SE, Hurley TG, Hussey JR, Hébert JR. Designing and developing a literature-derived, population-based dietary inflammatory index. Public Health Nutr. 2014;17:1689–96. 10.1017/s1368980013002115. 23941862 10.1017/S1368980013002115 PMC3925198

[32] Park YM, Shivappa N, Petimar J, Hodgson ME, Nichols HB, Steck SE, Hébert JR, Sandler DP. Dietary inflammatory potential, oxidative balance score, and risk of breast cancer: Findings from the Sister Study. Int J Cancer. 2021;149:615–26. 10.1002/ijc.33581. 33783833 10.1002/ijc.33581 PMC8256885

[33] Wu S, Zhu J, Lyu S, Wang J, Shao X, Zhang H, Zhong Z, Liu H, Zheng L, Chen Y. Impact of DNA-Methylation Age Acceleration on Long‐Term Mortality Among US Adults With Cardiovascular‐Kidney‐Metabolic Syndrome. J Am Heart Association. 2025;14:e039751. 10.1161/JAHA.124.039751 PMC12132836 40118808

[34] Wu S, Zhong Z, Wang Y, Wang J, Lyu S, Liu H, Chen Y. Unveiling the impact of DNA-methylation age acceleration on mortality risk in diabetes and pre-diabetes: insights from the US NHANES program. Clin Epigenetics. 2025;17:81. 10.1186/s13148-025-01886-0. 40380284 10.1186/s13148-025-01886-0 PMC12083022

[35] Galkin F, Kovalchuk O, Koldasbayeva D, Zhavoronkov A, Bischof E. Stress, diet, exercise: Common environmental factors and their impact on epigenetic age. Ageing Res Rev. 2023;88:101956. 10.1016/j.arr.2023.101956. 37211319 10.1016/j.arr.2023.101956

[36] Bordoni L, Agostinho de Sousa J, Zhuo J, von Meyenn F. Evaluating the connection between diet quality, EpiNutrient intake and epigenetic age: an observational study. Am J Clin Nutr. 2024;120:1143–55. 10.1016/j.ajcnut.2024.08.033. 39510725 10.1016/j.ajcnut.2024.08.033

[37] Kim Y, Huan T, Joehanes R, McKeown NM, Horvath S, Levy D, Ma J. Higher diet quality relates to decelerated epigenetic aging. Am J Clin Nutr. 2022;115:163–70. 10.1093/ajcn/nqab201. 34134146 10.1093/ajcn/nqab201 PMC8755029

[38] Wang X, Sarker SK, Cheng L, Dang K, Hu J, Pan S, Zhang J, Xu X, Li Y. Association of dietary inflammatory potential, dietary oxidative balance score and biological aging. Clin Nutr. 2024;43:1–10. 10.1016/j.clnu.2023.11.007. 37992632 10.1016/j.clnu.2023.11.007

[39] Petermann-Rocha F, Wirth MD, Boonpor J, Parra-Soto S, Zhou Z, Mathers JC, Livingstone K, Forrest E, Pell JP, Ho FK, et al. Associations between an inflammatory diet index and severe non-alcoholic fatty liver disease: a prospective study of 171,544 UK Biobank participants. BMC Med. 2023;21. 10.1186/s12916-023-02793-y. 10.1186/s12916-023-02793-y PMC10071692 37013578

[40] Garcia-Arellano A, Martínez-González MA, Ramallal R, Salas-Salvadó J, Hébert JR, Corella D, Shivappa N, Forga L, Schröder H, Muñoz-Bravo C, et al. Dietary inflammatory index and all-cause mortality in large cohorts: The SUN and PREDIMED studies. Clin Nutr. 2019;38:1221–31. 10.1016/j.clnu.2018.05.003. 30651193 10.1016/j.clnu.2018.05.003

[41] Veronese N, Cisternino AM, Shivappa N, Hebert JR, Notarnicola M, Reddavide R, Inguaggiato R, Guerra V, Logroscino A, Rotolo O, et al. Dietary inflammatory index and mortality: a cohort longitudinal study in a Mediterranean area. J Hum Nutr dietetics: official J Br Diet Association. 2020;33:138–46. 10.1111/jhn.12701. 10.1111/jhn.12701 31829488

[42] Zhao C, Lin M, Yang Y, Yang H, Gao Z, Yan Z, Liu C, Yu S, Zhang Y. Association between dietary inflammatory index and cardiovascular-kidney-metabolic syndrome risk: a cross-sectional study. Nutr J. 2025;24:60. 10.1186/s12937-025-01127-3. 40221720 10.1186/s12937-025-01127-3 PMC11992876

[43] Yu H, Liu Y, Zhang T, Guan Z, Li P. Association between dietary inflammatory index score and cardiovascular-kidney-metabolic syndrome: a cross-sectional study based on NHANES. Front Nutr. 2025;12:1557491. 10.3389/fnut.2025.1557491. 40416382 10.3389/fnut.2025.1557491 PMC12098081

[44] Bao W, Liu C, Huang W, Lai Y, Chen F, Yao Y, Lin HC, Ye Z, Qian J, Ping F, et al. Dietary inflammatory potential and dietary quality in relation to advanced cardiovascular-kidney-metabolic syndrome and mortality risk: traditional and machine learning-based analysis. Nutr J. 2026;25. 10.1186/s12937-025-01268-5. 10.1186/s12937-025-01268-5 PMC12870726 41495784

[45] López-Otín C, Blasco MA, Partridge L, Serrano M, Kroemer G. Hallmarks of aging: An expanding universe. Cell. 2023;186:243–78. 10.1016/j.cell.2022.11.001. 36599349 10.1016/j.cell.2022.11.001

[46] Furman D, Campisi J, Verdin E, Carrera-Bastos P, Targ S, Franceschi C, Ferrucci L, Gilroy DW, Fasano A, Miller GW, et al. Chronic inflammation in the etiology of disease across the life span. Nat Med. 2019;25:1822–32. 10.1038/s41591-019-0675-0. 31806905 10.1038/s41591-019-0675-0 PMC7147972

[47] Evans LW, Stratton MS, Ferguson BS. Dietary natural products as epigenetic modifiers in aging-associated inflammation and disease. Nat Prod Rep. 2020;37:653–76. 10.1039/c9np00057g. 31993614 10.1039/c9np00057g PMC7577396

[48] Nani A, Murtaza B, Sayed Khan A, Khan NA, Hichami A. Antioxidant and Anti-Inflammatory Potential of Polyphenols Contained in Mediterranean Diet in Obesity: Molecular Mechanisms. Molecules. 2021;26. 10.3390/molecules26040985. 10.3390/molecules26040985 PMC7918790 33673390

[49] Lu AT, Quach A, Wilson JG, Reiner AP, Aviv A, Raj K, Hou L, Baccarelli AA, Li Y, Stewart JD, et al. DNA methylation GrimAge strongly predicts lifespan and healthspan. Aging. 2019;11:303–27. 10.18632/aging.101684. 30669119 10.18632/aging.101684 PMC6366976

[50] Belsky DW, Caspi A, Corcoran DL, Sugden K, Poulton R, Arseneault L, Baccarelli A, Chamarti K, Gao X, Hannon E, et al. DunedinPACE, a DNA methylation biomarker of the pace of aging. eLife. 2022;11. 10.7554/eLife.73420. 10.7554/eLife.73420 PMC8853656 35029144

[51] Yang Z, Li Y, Liu Y, Zhong Z, Ditchfield C, Guo T, Yang M, Chen Y. Prognostic effects of glycaemic variability on diastolic heart failure and type 2 diabetes mellitus: insights and 1-year mortality machine learning prediction model. Diabetol Metab Syndr. 2024;16. 10.1186/s13098-024-01534-2. 10.1186/s13098-024-01534-2 PMC11585110 39578908

[52] Krebs-Smith SM, Pannucci TE, Subar AF, Kirkpatrick SI, Lerman JL, Tooze JA, Wilson MM, Reedy J. Update of the Healthy Eating Index: HEI-2015. J Acad Nutr Diet. 2018;118:1591–602. 10.1016/j.jand.2018.05.021. 30146071 10.1016/j.jand.2018.05.021 PMC6719291

