# Supplementary material for: Epigenetic age acceleration mediates the association between pro-inflammatory and pro-oxidant diets and the progression and mortality of cardiovascular-kidney-metabolic syndrome
Source: Genes Nutr. 2026 May 7;21:14. doi: 10.1186/s12263-026-00804-0 (PMC13321538; doi:10.1186/s12263-026-00804-0)
Supplement: Supplementary file 1 — Supplementary Material 1 [file 12263_2026_804_MOESM1_ESM.pdf]

eTable 1. Definitions of CKM

eTable 2. Detailed algorithm of the simplified 10-year CVD risk models

eTable 3. Detailed algorithm for evaluating each CKM stage

eTable 4. Dietary inflammatory index assignment scheme

eTable 5. Dietary Oxidative balance score assignment scheme

eTable 6. The components and scoring criteria of HEI-2015

eTable 7. Proportions of missing value

eTable 8. Sensitivity analysis of dietary indices and mortality outcomes in CKM patients after excluding patients died within the first two-year follow-up

eTable 9. Sensitivity analysis of dietary indices and mortality outcomes in CKM patients after excluding patients with cancer

eFigure 1. Flowchart of this study.

eTable 1. Definitions of CKM

CKM conditions	Definition	CKM indicators	Threshold for CKM indicators
CVD	Individuals with clinical CVD or subclinical CVD	Clinical CVD	History of chronic heart failure, coronary heart disease, heart attack, or stroke
		Subclinical CVD	Any of the following criterion is met:<br>1) Very high-risk CKD in KDIGO classification: UACR ≥ 300 mg/g and eGFR ≤ 45-59 ml/min/1.73m2, UACR ≥ 30 mg/g and eGFR ≤ 30-44 ml/min/1.73m2, or eGFR ≤ 29 ml/min/1.73m2.<br>2) Predicted 10-year CVD risk ≥ 20%
Kidney diseases	Individuals with CKD	CKD	Moderate-to-high-risk CKD in KDIGO classification: UACR ≥ 30 mg/g and eGFR ≥ 60 ml/min/1.73m2, UACR < 300 mg/g and eGFR ≤ 45-59 ml/min/1.73m2, or UACR < 30 mg/g and eGFR ≤ 30-44 ml/min/1.73m2.
Metabolic disorders	Individuals with overweight/obesity, abdominal obesity, prediabetes, diabetes, hypertension, hypertriglyceridemia or MetS	Overweight/obesity	BMI ≥25 kg/m2 (or ≥23 kg/m2 if Asian ancestry) *
		Abdominal obesity	Waist circumference ≥88/102 cm in female/male (or if Asian ancestry ≥80/90 cm in female/male)
		Prediabetes	Fasting blood glucose ≥ 100-124 mg/dL or HbA1c ≥ 5.7%-6.4% and without self-reported diagnosis of diabetes, use of insulin, or oral hypoglycemic agents
		Diabetes	Fasting blood glucose ≥ 125 mg/dL or HbA1c ≥ 6.5% or self-reported diagnosis of diabetes, use of insulin, or oral hypoglycemic agents
		Hypertension	SBP ≥130 mm Hg or DBP ≥80 mm Hg or self-reported diagnosis of hypertension or use of antihypertensive medications
		Hypertriglyceridemia	Triglycerides ≥ 135 mg/dL
		MetS	MetS is defined by the presence of 3 or more of the following:<br>1) Waist circumference ≥88/102 cm in female/male (or if Asian ancestry ≥80/90 cm in female/male).<br>2) HDL cholesterol <50/40 mg/dL in female/male.<br>3) Triglycerides ≥150 mg/dL.<br>4) Elevated blood pressure (SBP ≥130 mm Hg or DBP ≥80 mm Hg and/or use of antihypertensive medications)<br>5) Fasting blood glucose ≥100 mg/dL

*Asian was not listed as a separate race/ethnicity until NAHNES 2011-2012, therefore the uniform threshold for BMI and waist circumference was used in all participants in NHANES 1999-2010.

Abbreviations: BMI = body mass index; CKD = chronic kidney disease; CKM = cardiovascular-kidney-metabolic syndrom; CVD = cardiovascular disease; DBP = diastolic blood pressure; eGFR = estimated glomerular filtration rate; HDL-C = high-density lipoprotein cholesterol; KDIGO = The Kidney Disease: Improving Global Outcomes; MetS = metabolic syndrome; SBP = systolic blood pressure; UACR = urinary albumin to creatinine ratio.

eTable 2. Detailed algorithm of the simplified 10-year cardiovascular disease risk models

Sex	Calculation
Women	log-Odds = -3.307728 + 0.7939329 × (age – 55) /10 + 0.0305239 × (TC – HDL-C – 3.5) – 0.1606857 × (HDL-C – 1.3) /0.3 – 0.2394003 × (min(SBP, 110) – 110) /20 + 0.360078 × (max(SBP, 110) – 130) /20 + 0.8667604 × (if diabetes) + 0.5360739 × (if current smoker) + 0.6045917 × (min(eGFR, 60) – 60) / -15 + 0.0433769 × (max(eGFR, 60) – 90) / -15 + 0.3151672 × (if using anti-hypertensive medication) – 0.1477655 × (if using statin) – 0.0663612 × (if using anti-hypertensive medication) × (max(SBP, 110) – 130) /20 + 0.1197879 × (if using statin) × (TC – HDL-C – 3.5) – 0.0819715 × (age – 55) /10 × (TC – HDL-C – 3.5) + 0.0306769 × (age – 55) /10 × (HDL-C – 1.3) /0.3 – 0.0946348 × (age – 55) /10 × (max(SBP, 110) – 130) /20 – 0.27057 × (age – 55) /10 × (if diabetes) – 0.078715 × (age – 55) /10 × (if current smoker) – 0.1637806 × (age – 55) /10 × (min(eGFR, 60) – 60) / -15<br>Risk = exp(log-Odds) / (1 + exp(log-Odds))
Men	log-Odds = -3.031168 + 0.7688528 × (age – 55) /10 + 0.0736174 × (TC – HDL-C – 3.5) – 0.0954431 × (HDL-C – 1.3) /0.3 – 0.4347345 × (min(SBP, 110) – 110) /20 + 0.3362658 × (max(SBP, 110) – 130) /20 + 0.7692857 × (if diabetes) + 0.4386871 × (if current smoker) + 0.5378979 × (min(eGFR, 60) – 60) / -15 + 0.0164827 × (max(eGFR, 60) – 90) / -15 + 0.288879 × (if using anti-hypertensive medication) – 0.1337349 × (if using statin) – 0.0475924 × (if using anti-hypertensive medication) × (max(SBP, 110) – 130) /20 + 0.150273 × (if using statin) × (TC – HDL-C – 3.5) – 0.0517874 × (age – 55) /10 × (TC – HDL-C – 3.5) + 0.0191169 × (age – 55) /10 × (HDL-C – 1.3) /0.3 – 0.1049477 × (age – 55) /10 × (max(SBP, 110) – 130) /20 – 0.2251948 × (age – 55) /10 × (if diabetes) – 0.0895067 × (age – 55) /10 × (if current smoker) – 0.1543702 × (age – 55) /10 × (min(eGFR, 60) – 60) / -15<br>Risk = exp(log-Odds) / (1 + exp(log-Odds))

Abbreviations: eGFR = estimated glomerular filtration rate; HDL-C = high-density lipoprotein cholesterol; SBP = systolic blood pressure; TC = total cholesterol.

eTable 3. Detailed algorithm for evaluating each CKM stage

CKM stages	Definition	Criterion	Threshold for CKM conditions
Stage 0: No CKM risk factors	Individuals with normal BMI and waist circumference, normoglycemia, normotension, a normal lipid profile, and no evidence of CKD or subclinical or clinical CVD	All criteria are met	BMI <25 kg/m^2 (or <23 kg/m^2 if Asian ancestry)
			Waist circumference <88/102 cm in female/male (or if Asian ancestry <80/90 cm in female/male)
			Fasting blood glucose < 100 mg/dL and HbA1c < 5.7% and without self-reported diagnosis of diabetes, use of insulin, or oral hypoglycemic agents
			SBP <130 mm Hg and DBP <80 mm Hg without self-reported diagnosis of hypertension or use of antihypertensive medications
			HDL cholesterol >50/40 mg/dL in female/male and triglycerides < 150 mg/dL
			Low-risk CKD in KDIGO classification according to eGFR and UACR: UACR < 30 mg/g and eGFR ≥ 60 ml/min/1.73m^2.
			Predicted 10-year CVD risk < 20%
			No clinical CVD
Stage 1: Excess or dysfunctional adiposity	Individuals with overweight/obesity, abdominal obesity, or dysfunctional adipose tissue, without the presence of other metabolic risk factors or CKD	Any of the three criteria is met	Overweight/obesity
			Abdominal obesity
			Prediabetes
		All criteria are met	SBP <130 mm Hg and DBP <80 mm Hg without self-reported diagnosis of hypertension or use of antihypertensive medications
			HDL cholesterol >50/40 mg/dL in female/male and triglycerides <150 mg/dL
			Low-risk CKD in KDIGO classification according to eGFR and UACR: UACR < 30 mg/g and eGFR ≥ 60 ml/min/1.73m^2
			Predicted 10-year CVD risk < 20%
			No clinical CVD
Stage 2: Metabolic risk factors and CKD	Individuals with metabolic risk factors (hypertriglyceridemia, hypertension, MetS, diabetes), or CKD	Any of the five criteria is met	Hypertriglyceridemia
			Hypertension
			diabetes
			MetS
			Moderate-to-high-risk CKD in KDIGO classification
		All criteria are met	No very high-risk CKD in KDIGO classification
			Predicted 10-year CVD risk < 20%
			No clinical CVD
Stage 3: Subclinical CVD in CKM	Subclinical CVD among individuals with excess/dysfunctional adiposity, other	Any of the two criteria is met	Very high-risk CKD in KDIGO classification
			Predicted 10-year CVD risk ≥ 20%
		Any of the eight criteria is met	Overweight/obesity
			Abdominal obesity

	metabolic risk factors, or CKD		Prediabetes
			Hypertriglyceridemia
			Hypertension
			diabetes
			MetS
			Moderate-to-high-risk CKD in KDIGO classification
		The criterion is met	No clinical CVD
Stage 4: Clinical CVD in CKM	Clinical CVD among individuals with excess/dysfunctional adiposity, other metabolic risk factors, or CKD	The criterion is met	Clinical CVD
		Any of the nine criteria is met	Overweight/obesity
			Abdominal obesity
			Prediabetes
			Hypertriglyceridemia
			Hypertension
			diabetes
			MetS
			Moderate-to-high-risk CKD in KDIGO classification
			Very high-risk CKD in KDIGO classification

Abbreviations: BMI = body mass index; CKD = chronic kidney disease; CKM = cardiovascular-kidney-metabolic syndrom; CVD = cardiovascular disease; DBP = diastolic blood pressure; eGFR = estimated glomerular filtration rate; HDL-C = high-density lipoprotein; KDIGO = The Kidney Disease: Improving Global Outcomes; NHANES = National Health and Nutrition Examination Survey; SBP = systolic blood pressure; UACR = urinary albumin to creatinine ratio.

eTable 4. Dietary inflammatory index assignment scheme

Food Parameter	Unit	Overall Inflammatory Effect	Direction of Effect
**Pro-inflammatory Parameters**			
Carbohydrate	g	0.097	+
Cholesterol	mg	0.110	+
Energy	kcal	0.180	+
Total Fat	g	0.298	+
Iron (Fe)	mg	0.032	+
Saturated Fat	g	0.373	+
Trans Fat	g	0.229	+
Vitamin B12	µg	0.106	+
**Anti-inflammatory Parameters**			
Alcohol	g	-0.278	-
Beta-Carotene	µg	-0.584	-
Caffeine	g	-0.110	-
Eugenol	mg	-0.140	-
Fiber	g	-0.663	-
Folic Acid	µg	-0.190	-
Garlic	g	-0.412	-
Ginger	g	-0.453	-
Flavan-3-ol	mg	-0.415	-
Flavones	mg	-0.616	-
Flavonols	mg	-0.467	-
Flavanones	mg	-0.250	-
Anthocyanidins	mg	-0.131	-
Isoflavones	mg	-0.593	-

Green/Black Tea	g	-0.536	-
Magnesium (Mg)	mg	-0.484	-
Monounsaturated Fat (MUFA)	g	-0.009	-
Niacin	mg	-0.246	-
n-3 Fatty Acids	g	-0.436	-
n-6 Fatty Acids	g	-0.159	-
Onion	g	-0.301	-
Pepper	g	-0.131	-
Polyunsaturated Fat (PUFA)	g	-0.337	-
Riboflavin	mg	-0.068	-
Saffron	g	-0.140	-
Selenium (Se)	µg	-0.191	-
Thiamin	mg	-0.098	-
Thyme/Oregano	mg	-0.102	-
Turmeric	mg	-0.785	-
Vitamin A	RE	-0.401	-
Vitamin B6	mg	-0.365	-
Vitamin C	mg	-0.424	-
Vitamin D	µg	-0.446	-
Vitamin E	mg	-0.419	-
Zinc (Zn)	mg	-0.313	-
Rosemary	mg	-0.013	-
**Neutral/Very Weak Effect**			
Protein	g	0.021	+

Abbreviations: g = gram; mg = milligram; µ g = microgram; kcal = kilocalorie; RE = Retinol Equivalents; Fe = Iron; MUFA = Monounsaturated Fatty Acids; PUFA = Polyunsaturated Fatty Acids; Mg = Magnesium; Se = Selenium; Zn = Zinc.

eTable 5. Dietary oxidative balance score assignment scheme

DOBS components	Property	Male			Female		
		0	1	2	0	1	2
Dietary fiber (g/d)	Antioxidant	<14.25	14.25–21.20	≥21.20	<12.25	12.25–18.00	≥18.00
β-Carotene (RE/d)	Antioxidant	<710.00	710.00–2178.50	≥2178.50	<839.50	839.50–2371.00	≥2371.00
Vitamin B2 (mg/d)	Antioxidant	<2.06	2.06–3.41	≥3.41	<1.68	1.68–3.04	≥3.04
Niacin (mg/d)	Antioxidant	<24.82	24.82–40.32	≥40.32	<18.64	18.64–33.92	≥33.92
Vitamin B6 (mg/d)	Antioxidant	<1.67	1.67–2.39	≥2.39	<1.31	1.31–1.90	≥1.90
Total folate (mcg/d)	Antioxidant	<389.50	389.50–903.50	≥903.50	<322.00	322.00–938.50	≥938.5
Vitamin B12 (mcg/d)	Antioxidant	<4.62	4.62–15.14	≥15.14	<3.71	3.71–15.33	≥15.33
Vitamin C (mg/d)	Antioxidant	<39.25	39.25–109.935	≥109.935	<40.41	40.41–115.32	≥115.32
Vitamin E (ATE) (mg/d)	Antioxidant	<6.20	6.20–9.49	≥9.49	<5.25	5.25–8.24	≥8.24
Calcium (mg/d)	Antioxidant	<771.50	771.50–1219.00	≥1219.00	<802.50	802.50–1362.00	≥1362.00
Magnesium (mg/d)	Antioxidant	<274.00	274.00–386.00	≥386.00	<234.00	234.00–325.50	≥325.50
Zinc (mg/d)	Antioxidant	<11.12	11.12–19.73	≥19.73	<8.58	8.58–17.78	≥17.78
Copper (mg/d)	Antioxidant	<1.18	1.18–1.85	≥1.85	<1.02	1.02–1.62	≥1.62
Selenium (mcg/d)	Antioxidant	<108.10	108.10–164.20	≥164.20	<81.90	81.90–124.90	≥124.90
Total fat (g/d)	Prooxidant	≥90.18	61.50–90.18	<61.50	≥71.90	47.26–71.90	<47.26
Iron (mg/d)	Prooxidant	≥19.70	12.66–19.70	<12.66	≥16.99	10.65–16.99	<10.65

Abbreviations: DOBS = Dietary Oxidative Balance Score; g/d = grams per day; mg/d = milligrams per day; μg/d or mcg/d = micrograms per day; RE/d = Retinol Equivalents per day; ATE = alpha-Tocopherol Equivalents.

eTable 6. The components and scoring criteria of HEI-2015

HEI Components	Range of Points	Minimum Scoring Standard	Maximum Scoring Standard
Adequacy Components (higher score indicates higher consumption)			
Total Fruits	0-5	0	0.8 cup equiv. /1000 kcal
Whole Fruits	0-5	0	0.4 cup equiv./1000 kcal
Total Vegetables	0-5	0	1.1 cup equiv. /1000 kcal
Greens and Beans	0-5	0	0.2 cup equiv. /1000 kcal
Total Protein Foods	0-5	0	2.5 oz equiv./1000 kcal
Seafood and Plant Proteins	0-5	0	0.8 oz equiv./1000 kcal
Dairy	0-10	0	1.3 cup equiv./1000 kcal
Whole Grains	0-10	0	1.5 oz equiv. /1000 kcal
Fatty Acids [b]	0-10	(PUFAs + MUFAs)/SFAs ≤1.2	(PUFAs + MUFAs)/SFAs ≥2.5
Moderation Components (higher score indicates lower consumption)			
Refined Grains	0-10	4.3 oz equiv./1000 kcal	1.8 oz equiv. /1000 kcal
Sodium	0-10	2.0 grams /1000 kcal	1.1 grams/1000 kcal
Added Sugars	0-10	26% of energy	6.5% of energy
Saturated Fats	0-10	16% of energy	8% of energy

[a]Intakes between the minimum and maximum standards are scored proportionately.

[b]Ratios of polyunsaturated and monounsaturated fatty acids (PUFAs and MUFAs) to saturated fatty acids (SFAs).

Abbreviations: HEI = Healthy Eating Index; PUFAs = polyunsaturated monounsaturated fatty acids; MUFAs = monounsaturated fatty acids; SFAs = saturated fatty acids.

eTable 7. Proportions of missing value

Characteristics	Overall participants (n = 2109)	
	N	Percentage, %
Poverty income ratio	235/2109	11.14
Smoking status	4/2109	0.19
Body mass index	78/2109	3.70
Systolic blood pressure	11/2109	0.52
Physical activity	1/2109	0.05
Hemoglobin A1c	1/2109	0.05
Estimated glomerular filtration rate	1/2109	0.05
High-density lipoprotein cholesterol	1/2109	0.05

eTable 8. Sensitivity analysis of dietary indices and mortality outcomes in CKM patients after excluding patients died within the first two-year follow-up

	All-cause mortality		Cardiovascular mortality		Non-cardiovascular mortality	
	**HR (95% CI)**	**P**	**HR (95% CI)**	**P**	**HR (95% CI)**	**P**
**Continuous DII**	1.07 (1.02, 1.12)	0.005	1.17 (1.07, 1.29)	< 0.001	1.04 (0.98, 1.09)	0.208
**Categorical DII**						
**Tertile 1 (< 0.87)**	*Reference*		*Reference*		*Reference*	
**Tertile 2 (0.87-2.38)**	1.10 (0.94, 1.30)	0.224	1.36 (0.99, 1.87)	0.061	1.03 (0.86, 1.24)	0.754
**Tertile 3 (≥ 2.38)**	1.28 (1.08, 1.53)	0.006	1.79 (1.26, 2.53)	0.001	1.15 (0.94, 1.41)	0.188
**P for trend**		0.005		0.001		0.180
**Continuous DOBS**	0.98 (0.97, 0.99)	0.002	0.96 (0.94, 0.98)	< 0.001	0.99 (0.99, 1.00)	0.113
**Categorical DOBS**						
**Tertile 1 (< 10)**	*Reference*		*Reference*		*Reference*	
**Tertile 2 (10-17)**	0.92 (0.80, 1.06)	0.254	0.78 (0.59, 1.04)	0.086	0.98 (0.83, 1.16)	0.784
**Tertile 3 (≥ 17)**	0.81 (0.69, 1.06)	0.011	0.65 (0.47, 0.91)	0.010	0.87 (0.72, 1.06)	0.158
**P for trend**		0.012		0.009		0.170
**Different combinations of DII and DOBS**						
**Pro-inflammatory and pro-oxidative diet**	*Reference*		*Reference*		*Reference*	
**Composite diet category**	0.83 (0.71, 0.96)	0.011	0.72 (0.55, 0.96)	0.025	0.87 (0.73, 1.03)	0.098
**Anti-inflammatory and antioxidant diet**	0.75 (0.62, 0.91)	0.004	0.62 (0.42, 0.91)	0.015	0.80 (0.64, 1.00)	0.052
**P for trend**		0.003		0.012		0.047

All models were adjusted for age, sex, race and ethnicity, poverty income ratio, smoking status, and alcohol consumption, physical activity, Healthy Eating Index-2015, C-reactive protein, and self-reported cancer.
Abbreviations: CKM, cardiovascular-kidney-metabolic syndrome; CI, confidence interval; DII, dietary inflammatory index; DOBS, dietary oxidative balance score; OR, odds ratio.

eTable 9. Sensitivity analysis of dietary indices and mortality outcomes in CKM patients after excluding patients with cancer

	All-cause mortality		Cardiovascular mortality		Non-cardiovascular mortality	
	**HR (95% CI)**	**P**	**HR (95% CI)**	**P**	**HR (95% CI)**	**P**
**Continuous DII**	1.08 (1.03, 1.13)	< 0.001	01.17 (1.07, 1.28)	< 0.001	1.05 (1.00, 1.11)	0.055
**Categorical DII**						
**Tertile 1 (< 0.87)**	*Reference*		*Reference*		*Reference*	
**Tertile 2 (0.87-2.38)**	1.11 (0.95, 1.30)	0.176	1.35 (0.99, 1.83)	0.056	1.04 (0.87, 1.25)	0.661
**Tertile 3 (≥ 2.38)**	1.34 (1.13, 1.59)	< 0.001	1.71 (1.23, 2.39)	0.002	1.24 (1.01, 1.51)	0.036
**P for trend**		< 0.001		0.001		0.032
**Continuous DOBS**	0.98 (0.97, 0.99)	< 0.001	0.96 (0.94, 0.98)	0.002	0.99 (0.98, 1.00)	0.029
**Categorical DOBS**						
**Tertile 1 (< 10)**	*Reference*		*Reference*		*Reference*	
**Tertile 2 (10-17)**	0.90 (0.78, 1.03)	0.125	0.76 (0.58, 0.99)	0.043	0.96 (0.81, 1.13)	0.595
**Tertile 3 (≥ 17)**	0.77 (0.66, 0.90)	0.001	0.61 (0.45, 0.84)	0.002	0.84 (0.69, 1.01)	0.060
**P for trend**		0.001		0.002		0.065
**Different combinations of DII and DOBS**						
**Pro-inflammatory and pro-oxidative diet**	*Reference*		*Reference*		*Reference*	
[illegible]	[illegible]	[illegible]	[illegible]	[illegible]	[illegible]	[illegible]
**Anti-inflammatory and antioxidant diet**	0.70 (0.58, 0.84)	< 0.001	0.60 (0.42, 0.87)	0.006	0.74 (0.59, 0.92)	0.006
**P for trend**		< 0.001		0.005		0.005

All models were adjusted for age, sex, race and ethnicity, poverty income ratio, smoking status, and alcohol consumption, physical activity, Healthy Eating Index-2015, C-reactive protein, and self-reported cancer.

Abbreviations: CKM, cardiovascular-kidney-metabolic syndrome; CI, confidence interval; DII, dietary inflammatory index; DOBS, dietary oxidative balance score; OR, odds ratio.

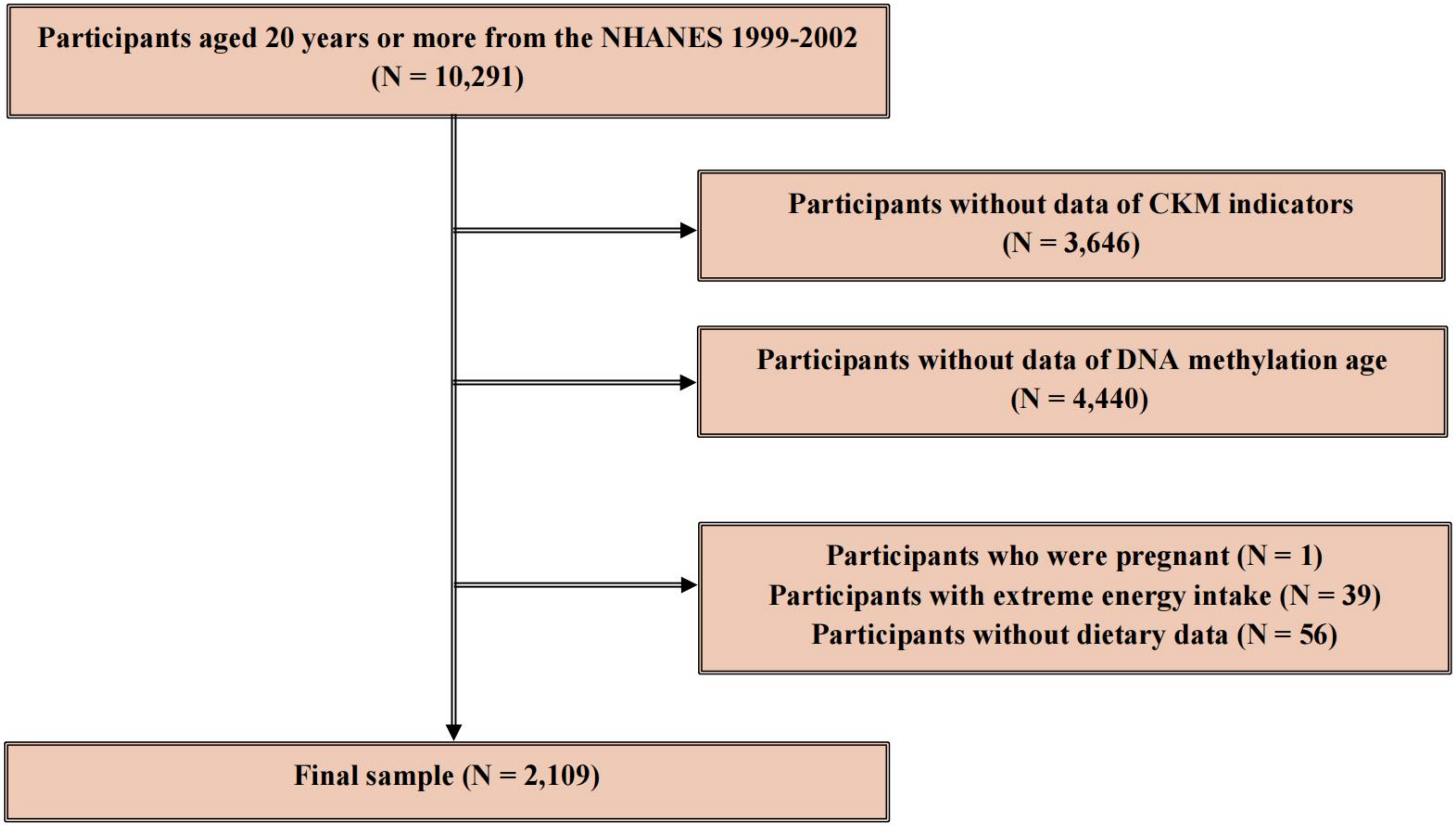

eFigure 1 Flowchart of this study.
Abbreviations: CKM, cardiovascular-kidney-metabolic; NHANES, National Health and Nutrition Examination Survey.
